# Two Strategies of *Pseudomonas syringae* to Avoid Recognition of the HopQ1 Effector in *Nicotiana* Species

**DOI:** 10.3389/fpls.2018.00978

**Published:** 2018-07-10

**Authors:** Patrycja Zembek, Aleksandra Danilecka, Rafał Hoser, Lennart Eschen-Lippold, Marta Benicka, Marta Grech-Baran, Wojciech Rymaszewski, Izabela Barymow-Filoniuk, Karolina Morgiewicz, Jakub Kwiatkowski, Marcin Piechocki, Jaroslaw Poznanski, Justin Lee, Jacek Hennig, Magdalena Krzymowska

**Affiliations:** ^1^Institute of Biochemistry and Biophysics (PAS), Warsaw, Poland; ^2^Leibniz Institute of Plant Biochemistry, Halle, Germany

**Keywords:** TTSS effectors, HopQ1, HopR1, virulence, *Pseudomonas syringae*

## Abstract

*Pseudomonas syringae* employs a battery of type three secretion effectors to subvert plant immune responses. In turn, plants have developed receptors that recognize some of the bacterial effectors. Two strain-specific HopQ1 effector variants (for Hrp outer protein Q) from the pathovars *phaseolicola* 1448A (*Pph*) and *tomato* DC3000 (*Pto*) showed considerable differences in their ability to evoke disease symptoms in *Nicotiana benthamiana*. Surprisingly, the variants differ by only six amino acids located mostly in the N-terminal disordered region of HopQ1. We found that the presence of serine 87 and leucine 91 renders *Pto*HopQ1 susceptible to N-terminal processing by plant proteases. Substitutions at these two positions did not strongly affect *Pto*HopQ1 virulence properties in a susceptible host but they reduced bacterial growth and accelerated onset of cell death in a resistant host, suggesting that N-terminal mutations rendered *Pto*HopQ1 susceptible to processing *in planta* and, thus, represent a mechanism of recognition avoidance. Furthermore, we found that co-expression of HopR1, another effector encoded within the same gene cluster masks HopQ1 recognition in a strain-dependent manner. Together, these data suggest that HopQ1 is under high host-pathogen co-evolutionary selection pressure and *P. syringae* may have evolved differential effector processing or masking as two independent strategies to evade HopQ1 recognition, thus revealing another level of complexity in plant – microbe interactions.

## Introduction

*Pseudomonas syringae* is a widespread bacterium that can infect almost 200 plant species. Its various pathovars cause diseases in several agriculturally important plants – halo blight in bean, bacterial speck in tomato, bacterial blight in soybean or broccoli, angular leaf spot in cucumber or wildfire in tobacco. Like many other gram-negative pathogenic bacteria, *P. syringae* secretes type III effectors into host cells to facilitate colonization of plants. The effectors play multiple roles during the infection process. They are primarily used to subvert the host cellular machinery, but they are also involved in nutrient acquisition or control of microbial community (Snelders et al., 2018). Nearly 100 effector families have so far been identified in *P. syringae* (Büttner, 2016), however, the effector repertoire (effectome) of a particular strain does not usually exceed 30 proteins (Baltrus et al., 2011). Even a single effector may define the host range by promoting bacterial multiplication in one plant while in other species presence of this same effector may trigger plant defense response leading to cessation of bacterial growth. Thereby, the composition of the effectome contributes to host specificity of a given bacterial strain. Various forces shape the effectome but the most significant is the need to avoid plant recognition (Koebnik and Lindeberg, 2011). Several mechanisms to overcome selection pressure have been described for individual effectors including their loss, mutagenesis or acquisition of novel domains but the mechanisms that tailor the whole effectomes remain largely unknown (Koebnik and Lindeberg, 2011). Recent reports show (Wei et al., 2015, 2018) that interplay between effectors contributes to several aspects of the infection process including bacterial growth rate in plant tissues, symptom development but also suppression of host defense. The fact that one effector is able to suppress response triggered by the second effector from the cooperating pair suggests that adaptation to the partner may be another factor that drives evolution of effectors.

HopQ1 (for Hrp outer protein Q) is an effector hypothesized to be acquired recently by *P. syringae* (Rohmer et al., 2004). It promotes disease development in bean, tomato, and Arabidopsis plants (Ferrante et al., 2009; Li et al., 2013b). In contrast, HopQ1 is recognized by *Nicotiana* spp., which have evolved systems to sense its presence and initiate defense responses (Wei et al., 2007). This response is mediated by Roq1 (for Recognition of XopQ 1), a receptor that directly interacts with HopQ1 and XopQ, a close homolog from *Xanthomonas* spp. Therefore, to avoid perception, strains of *P. syringae* pv. *tabaci* evolutionarily eliminated the sequence encoding HopQ1 from their genomes (Ferrante et al., 2009). Here, we report two mechanisms employed by *P. syringae* to remain undetected in *Nicotiana* spp. despite expressing HopQ1.

## Materials and Methods

### *P. syringae* Strains and Inoculation

Sequences encoding HopQ1 from *P. syringae* pv. *tomato* DC3000 (*Pto*HopQ1), HopR1 from *P. syringae* pv. *phaseolicola* 1448A (*Pph*HopR1) or from *tomato* DC3000 (*Pto*HopR1) were PCR amplified (see Supplementary Tables 1, 2 for the list of the strains and primers used in this study) and cloned into the pENTR/D-TOPO vector. *hopQ1* variants were made by site-directed mutagenesis, as described previously (Giska et al., 2013). All the sequences were PCR amplified to add appropriate restriction sites and cloned into pJET 1.2. Next, the sequences were cut with the restriction enzymes and cloned under the control of Tac promoter in pBBR1-MCS2-pTac, the modified broad-host-range vector pBBR1MCS-2 (Giska et al., 2013).

To prepare pseudo-operons that co-express HopQ1 and HopR1, *hopR1* variants were PCR amplified with primers adding a ribosome binding site and a FLAG-epitope encoding sequence to the 5′ and 3′ ends of the products, respectively, and KpnI restriction sites to both ends. PCR products were cloned into pJET 1.2 and re-cloned into pBBR1MCS-2-pTac derivatives carrying appropriate *hopQ1* sequences. All the constructs were electroporated into *P. syringae* pv. *syringae* B728a and *Pto*DC3000D28E *P. syringae* strains. The bacteria were prepared for inoculation as described previously (Krzymowska et al., 2007). Following centrifugation at 3,500 × *g* for 10 min, the pellet was washed once and resuspended in sterile 10 mM MgCl_2_. The bacterial suspension was adjusted to OD_600_ = 0.2 (that corresponds to approximately 10^8^ colony forming units [cfu]/ml) and further diluted, as indicated. Bacterial titers were checked by plating.

To assay bacterial growth *in Nicotiana benthamiana*, whole plants were dip-inoculated with *Pss* (culture density 10^6^ cfu/ml) expressing HopQ1 variants or the pseudo-operons. At the indicated time points, three 1 cm-diameter leaf disks were punched out, surface-sterilized with 70% ethanol for 1 min, rinsed with sterile water for 1 min and ground in 300 μl 10 mM MgCl_2_. Serial dilutions were plated on LB agar plates for bacteria enumeration. To assess the impact of HopQ1 variants on *Pss* growth in *Nicotiana tabacum* plants, the bacterial suspensions expressing the indicated variants were infiltrated into leaves and the bacteria were isolated at the indicated time points.

For assessment of hypersensitive response (HR) development in tobacco, the leaves were infiltrated using a needleless syringe with bacterial suspensions adjusted to approximately 10^8^ cfu/ml. *Pto*DC3000D28E (50 μl) was applied locally and to measure loss of cell membrane integrity whole tobacco leaves were infiltrated with *Pss* suspension.

### Transient Expression in Protoplasts

To express C-terminally HA-tagged HopQ1 variants in Arabidopsis protoplasts, the sequences encoding the effector variants were recombined into pUGW14 vector (Nakagawa et al., 2007). Protoplast isolation, transformation and elicitation with flg22 was performed as described previously (Yoo et al., 2007; Ranf et al., 2011). Activation of MAP kinases was assayed with antibodies directed against the phosphorylated activation loop (anti-*p*TE*p*Y; #9101 Cell Signaling, Tech.). Protein amounts detected by immunoassay were calculated as described by Imkampe et al. (2017). Luciferase reporter activity (p*NHL10*-LUC) was measured and normalized as described previously (Pecher et al., 2014).

### Ion Conductivity

At the indicated time points, eight leaf disks (1 cm diameter) were cut from infiltrated zones and floated abaxial side up on 5 ml milliQ water for 10 min at 18°C with gyratory agitation (50 rpm). The conductivity of the water was measured with a WTW InoLab Multi 9310 IDSCDM83 benchtop meter and expressed in μScm^-1^.

### Confocal Laser Scanning Microscopy

To generate a construct expressing HopR1-eYFP, the entry clones carrying HopR1 variants were LR recombined with the Gateway pGWB441 destination vector. The resulting constructs were electroporated into *Agrobacterium tumefaciens* (GV3101) cells. Subsequently, *A. tumefaciens* cultures containing the constructs were infiltrated into *N. benthamiana* leaves, and tissues were analyzed using an FV1000 confocal system (Olympus, Tokyo, Japan) equipped with a 60x/1.2 water immersion objective lens. eYFP was excited with the 515 nm line from an argon ion laser and fluorescence signals were recorded using diffraction grate based spectral detector with 530–640 nm detection window. Chlorophyll autofluorescence was excited with 440 nm laser diode and detected using 750/50 emission filter (Chroma).

### Accession Numbers

Sequence data from this article can be found in the GenBank data libraries under accession numbers *Pph*HopQ1 (AAZ37975.1), *Pto*HopQ1 (also known as HopQ1-1, NP_790716.1), *Pph*HopR1 (AAZ37024.1), *Pto*HopR1 (NP_790722.1).

## Results and Discussion

**FIGURE 1:**
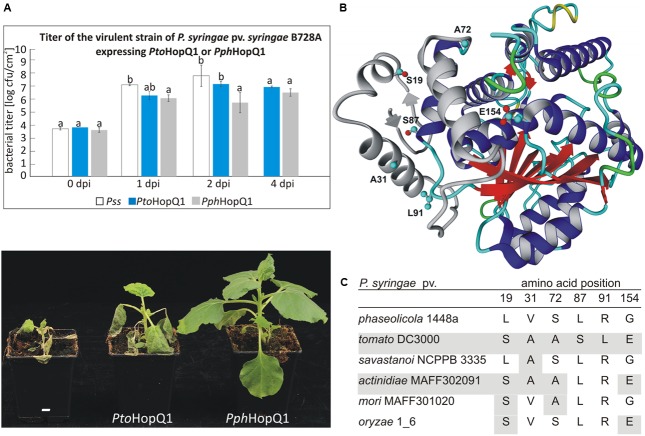
*Pto*HopQ1 and *Pph*HopQ1 differ in their avirulence properties. **(A)** *Nicotiana benthamiana* plants were dip-inoculated with *Pseudomonas syringae* pv. *syringae* B728A expressing either one of the effectors. Bacterial titers were determined at 0/1/2/4 days post inoculation (upper panel). Note that due to severe tissue collapse of *Pss* infected leaves, the collection of samples was not possible at 4 dpi. Lower panel shows the plants 7 days after inoculation. The experiment was performed three times with similar results. Data were analyzed using repeated measures analysis of variance (ANOVA), followed by Tukey HSD *post hoc* test performed for each time point separately. Statistically distinct groups are marked with different letters above each column. **(B)** Representative model of *Pto*HopQ1 generated by I-TASSER (Zhang, 2008; Roy et al., 2010) and visualized using Yasara View (Krieger and Vriend, 2014). The flexible N-terminal part of the protein, which varies in particular models, is shown in gray. The residues that differ in *Pto*HopQ1 compared to *Pph*HopQ1 are denoted in ball and stick representation. **(C)** Comparison of HopQ1 variants from the selected strains.

Despite a very high level of amino acid (aa) identity between two HopQ1 variants derived from *P. syringae* pv. *phaseolicola* 1448a (*Pph*) and *P. syringae* pv. *tomato* DC3000 (*Pto*), their expression in a virulent *P. syringae* strain resulted in different disease outcomes in dip-inoculated *N. benthamiana* plants (**Figure 1A**). Consistent with our previous experiments (Giska et al., 2013), *PphhopQ1* rendered *P. syringae* pv. *syringae* B728a (*Pss*) avirulent toward *N. benthamiana.* Introduction of *PtohopQ1* to *Pss* also reduced disease severity of *Pss* but compared to bacteria expressing *PphhopQ1*, the bacteria multiplied more rapidly at the early stages of the infection and evoked severe disease symptoms.

Since *Pto*HopQ1 and *Pph*HopQ1 proteins differ only in six aa (**Figure 1B**), we aimed at identification of those residues that affect the effector properties. To this end, we generated variants by site-directed mutagenesis. To reduce the number of possible variants, we focused on aa combinations that naturally occur in HopQ1 effectors in other *P. syringae* pathovars (**Figure 1C**), namely pv. *savastanoi* NCPPB3335, pv. *actinidiae* MAFF302091, pv. *mori* and pv. *oryzae* 1_6. Based on this sequence comparison, we prepared constructs encoding four *Pph*HopQ1 mutants (L19S; V31A; L19S_S72A; L19S_G154E) and one *Pto*HopQ1 mutant (S87L_L91R).

**FIGURE 2:**
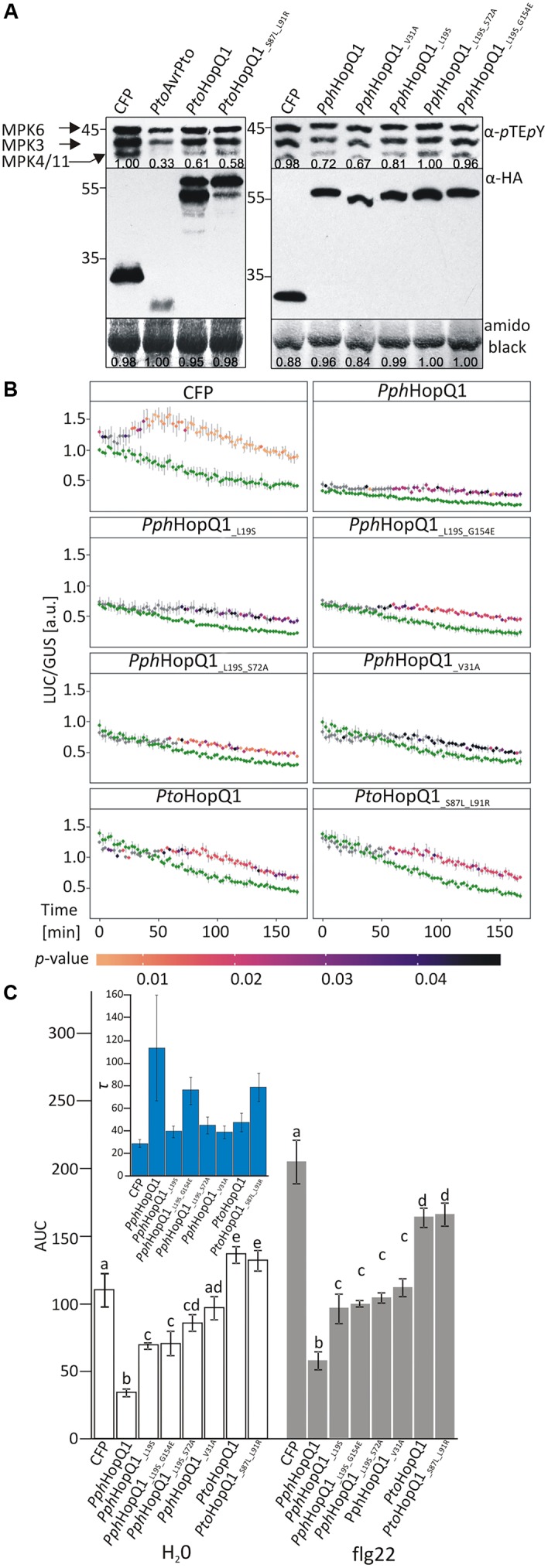
Effect of HopQ1 variants’ expression on defense-related MAPK activation. **(A)** Arabidopsis protoplasts were transformed with plasmids encoding hemagglutinin (HA)-tagged HopQ1 variants under the control of the cauliflower mosaic virus 35S promoter. Expression of cyan fluorescent protein (CFP) and AvrPto served as negative and positive controls, respectively. Fourteen hours after transformation, protoplasts were treated with 100 nM flg22 for 10 min. MAPK activation was monitored by immunoblot analysis with anti-*p*TE*p*Y antibodies and the expression level of HopQ1 variants was checked with anti-HA antibodies. Amido black staining of the membranes was used to demonstrate equal loading. The numbers correspond to ImageJ-based quantification of the protein band intensities (MAPK activation strength is the sum of all three MAPK bands). **(B)** Arabidopsis protoplasts were co-transformed with constructs expressing HopQ1 variants, *pNHL10-LUC* (luciferase) as a reporter and *pUBQ10-GUS* (β-glucuronidase). Luciferase activity was recorded for 3 h, following flg22 treatment, and depicted as LUC/GUS ratios. Data for each protein variant were analyzed using repeated measures ANOVA, yielding significant effects of variant, time and their interaction (*p* < 0.001). Differences between H_2_O-treated samples (green traces) and flg22 treatments were tested with Student’s *t*-test. Statistical significant differences in the flg22-treated samples as compared to the H_2_O-treated samples are highlighted by the color-coded *p*-values adjusted using Benjamini–Hochberg procedure. **(C)** Area under the curve (AUC) values were calculated for the graphs. One-way ANOVA was performed separately for both treatments and was followed by Tukey HSD *post hoc* test. Letters correspond to statistically homogenous groups (*p* < 0.05). Inlet: τ parameter values obtained after curve fitting to the fold changes for each protein variant (see Supplementary Figure 1). Bars correspond to standard errors in parameter estimation. The experiment was performed three times with similar results.

*Pto*HopQ1 has been reported previously to suppress flg22-induced activation of MAP kinases in Arabidopsis (Hann et al., 2014). Therefore, to assess the properties of the HopQ1 variants, we transiently expressed them in Arabidopsis mesophyll protoplasts. Both *Pto*HopQ1 variants equally suppressed flg22-mediated activation of MPK3, MPK6, and MPK4/11 in protoplasts (**Figure 2A**). For the *Pph*HopQ1 wild type and V31A mutant versions, also the same levels of suppression were observed. However, suppression of MAPK activation was less pronounced by the L19S single mutant and both double mutants, L19S-S72A and L19S-G154E, completely lost suppressive activity (**Figure 2A**).

To quantify the impact of HopQ1 variants on flg22-induced plant responses, we used a previously described luciferase reporter system that monitors expression of the firefly luciferase (*LUC*) gene under the control of the flg22-inducible *A. thaliana NHL10* (*NDR1/HIN1-LIKE 10*) promoter (Boudsocq et al., 2010; Pecher et al., 2014). In this assay, *Pph*HopQ1-WT strongly suppressed basal and flg22-induced *NHL10* promoter activity over a 3 h measurement period (**Figure 2B**). To facilitate comparison between the various HopQ1 variants and between treatments, total promoter activities were further calculated as “area under the curve” (AUC; **Figure 2C** and Supplementary Figure 1). All *Pph*HopQ1 mutant variants tested suppressed both basal and flg22-induced promoter activities, but were significantly less active than the WT version. Expression of both *Pto*HopQ1 variants enhanced basal promoter activity compared to the CFP transfection control (**Figure 2B**), leading to significantly higher total signal under control conditions (H_2_O; **Figure 2B**). Importantly, upon flg22 elicitation, *Pto*HopQ1-expressing samples did not reach the level of the CFP-expressing controls (**Figure 2B**) and total signal calculations revealed a significant reduction, indicative of a strong suppressive capability on elicitor-induced activity.

Interestingly, we noticed that *Pto*HopQ1, in contrast to *Pph*HopQ1, was reproducibly detected in two forms, presumably the full-length and a truncated version. Since the HA-tag was located at the C-terminus of *Pto*HopQ1, we could conclude that the truncated form of the effector was N-terminally cleaved. The presence of two forms of *Pto*HopQ1 was previously observed in transgenic Arabidopsis and tomato plants (Li et al., 2013a), as well as in *N. benthamiana* leaves transiently expressing *Pto*HopQ1 (Li et al., 2013b), suggesting that *Pto*HopQ1 is prone to N-terminal processing. Consistent with this notion, the six aa that are different in *Pto*HopQ1 compared to *Pph*HopQ1 lie within its N-terminus (**Figure 1B**). In contrast to the parental *Pto*HopQ1, the *Pto*HopQ1__S87L_L91R_ mutant was detected mainly in the presumed full-length form indicating that the presence of S87 and L91 renders *Pto*HopQ1 susceptible to proteolytic cleavage. These two aa are located in the predicted hinge region (loop) linking the N-terminal and the central nucleoside hydrolase domains, putatively an exposed area susceptible for cleavage (**Figure 1B**). ELM (Eukaryotic Linear Motif) analysis (Dinkel et al., 2016) revealed a region, located between aa 89 and 93, as a putative subtilisin cleavage site (Supplementary Figure 2) and subtilisin-like proteases (subtilases) are implicated in plant defense (Figueiredo et al., 2014). Although our data indicate that S87 and L91 are involved in N-terminal processing, they are not absolutely required since the S87L-L91R mutant still accumulates the truncated form but to a reduced extent. Consistently, reciprocal substitutions within *Pph*HopQ1 (*Pph*HopQ1__L87S_R91L_) lead to the partial cleavage of the effector indicating that although presence of these two aa renders HopQ1 susceptible to the cleavage it is not sufficient for the effective processing (Supplementary Figure 3). Importantly, the *Pto*HopQ1__S87L_L91R_ mutant showed a similar behavior like the wild type version in the luciferase reporter assay, thus, the cleavability of *Pto*HopQ1 can be uncoupled from the enhanced basal promoter activity as well as the suppression of flg22-induced promoter activity mediated by the effector. To further analyze the importance of *in planta Pto*HopQ1 processing for its virulence function, we concentrated our efforts on this aspect using the *Pto*HopQ1__S87L_L91R_ mutant as a tool.

**FIGURE 3:**
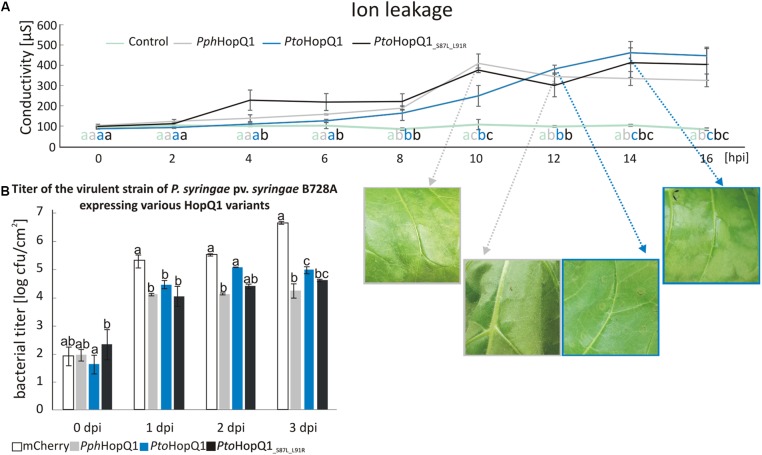
Presence of serine at the position 87 and leucine at the position 91 in HopQ1 sequence is associated with a delayed onset of HR and an increased bacterial growth rate in a resistant host. **(A)** Ion leakage assay. *Nicotiana tabacum* leaves were infiltrated with *Pss* bacteria (culture density ca. 10^8^ cfu/ml) expressing *Pph*HopQ1, *Pto*HopQ1, or *Pto*HopQ1__S87L_L91R_. At selected time points, cellular ion leakage to the apoplast was measured after floating leaf disks on the milliQ water. The photographs show leaf tissue at the time of visible symptoms development and/or at the maximal conductivity level. **(B)** *Pss* growth *in planta*. The bacterial suspensions (ca. 10^3^ cfu/ml) expressing the indicated variants were infiltrated into *N. tabacum* leaves and at the indicated time points bacteria were isolated and serial dilutions were plated for enumeration. Data were analyzed using repeated measures ANOVA, followed by Tukey HSD *post hoc* test performed for each time point separately. Letters correspond to statistically homogenous groups (*p* < 0.05). The experiment was performed twice with similar results.

Compared to patchy and non-homogenous necroses obtained with *N. benthamiana*, *N. tabacum* is a better model, for investigating HopQ1-triggered HR. It was previously shown that full-length HopQ1 triggers the HR in *N. tabacum* (Giska et al., 2013; Li et al., 2013a). Since expression of HopQ1 lacking the first 89 aa did not lead to visible tissue collapse (Li et al., 2013a), we hypothesized that the recognition of HopQ1 that undergoes cleavage may be compromised in tobacco plants. To test this, we introduced plasmids expressing *Pph*HopQ1, *Pto*HopQ1, and *Pto*HopQ1__S87L_L91R_ into *Pss* and measured bacteria-induced ion leakage that reflects loss of plasma membrane integrity in the course of the hypersensitive cell death (Krzymowska et al., 2007). As shown in **Figure 3A**, 10 h post infiltration (hpi), that is the time when first macroscopic signs of tissue collapse became visible, the conductivity reached the maximum level in response to *Pss* expressing *Pph*HopQ1. This effect was delayed in response to *Pto*HopQ1-expressing bacteria. In this case, the first symptoms – vitrification when viewed from the abaxial leaf surfaces – were only visible 12 hpi and the maximum increase in conductivity was reached at 14 hpi. Interestingly, upon infiltration of *Pss* expressing *Pto*HopQ1__S87L_L91R_, maximum conductivity was recorded already at 10 hpi but in contrast to *Pph*HopQ1, this level remained elevated till the last time point (16 hpi). These data suggest that the aa substitutions that reduce HopQ1 susceptibility to proteolytic cleavage may restore early recognition of the effector in tobacco plants. We asked further, whether the changed HopQ1 perception affects virulence of *Pss* in this host plant. Therefore, we monitored multiplication of the *Pss* strains expressing HopQ1 variants. As shown in **Figure 3B**, expression of all HopQ1, variants reduced the ability of *Pss* to grow in tobacco leaves compared to the mCherry-expressing control strain. This effect, however, was less pronounced with *Pss* expressing *Pto*HopQ1. This suggests that the delayed HR onset leads to an enhanced bacterial growth at the early stages of the infection and, thus, synthesis of the cleavable form of HopQ1 seems to be beneficial in the resistant host. Consistent with this model, bacteria expressing *Pto*HopQ1__S87L_L91R_ that is less prone to processing multiplied to intermediate levels.

**FIGURE 4:**
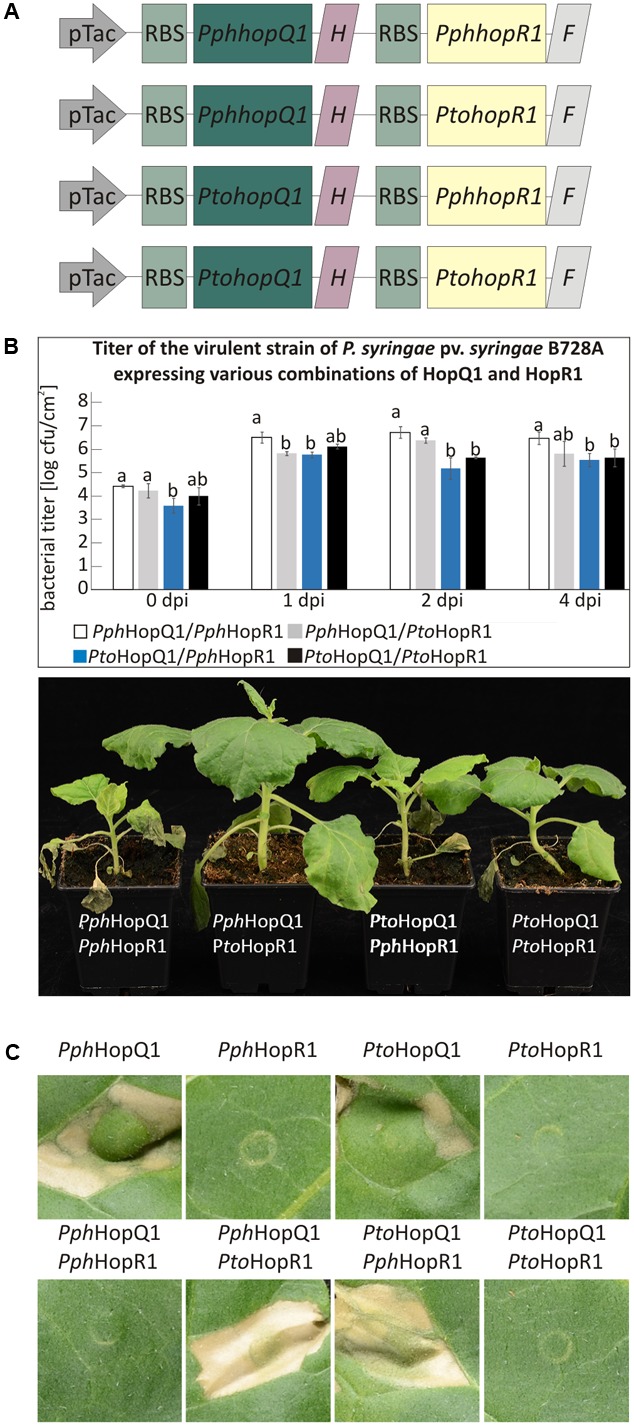
HopR1 masks HopQ1-mediated recognition of *P. syringae*. **(A)** A schematic representation of pseudo-operons that co-express HopQ1 and HopR1 from *P. syringae* pv. *phaseolicola* 1448A or tomato DC3000. ‘H’ and ‘F’ stands for His and FLAG tag, respectively. **(B)** *N. benthamiana* plants were dip-inoculated with *Pss* expressing the pseudo-operons. At the indicated time points bacteria were isolated from leaf tissue and serial dilutions were plated on LB agar plates. Data were analyzed using repeated measures ANOVA, followed by Tukey HSD *post hoc* test performed for each time point separately. Letters correspond to statistically homogenous groups (*p* < 0.05). The photographs were taken 7 days after inoculation. **(C)** *Pto*DC3000D28E strain expressing the indicated combinations of HopQ1 or HopR1 were locally infiltrated into *N. tabacum* leaves. Necrosis development was observed already 24 h later and the photographs were taken 5 days after infiltration. The experiment was performed twice with similar results.

Besides individual functions, effector proteins may act in concert within host cells and this is particularly likely for effectors that are sequentially delivered by the type III secretion system (Büttner, 2016). HopQ1 is grouped together with HopR1 in the same gene cluster in *Pto* (Kvitko et al., 2009). String database analysis^1^ revealed a significant co-occurrence of *hopQ1* with *hopR1*. These findings suggested that these two effectors might act co-operatively in plant cells. To test this hypothesis, we prepared vectors carrying pseudo-operons of *PtohopQ1* or *PphhopQ1* with *PtohopR1* or *PphhopR1* under control of a constitutive version of Tac promoter (**Figure 4A**). We introduced pseudo-operons carrying the sequences coding for the effector pairs into a *Pss* strain virulent on *N. benthamiana*. Subsequently, we scored disease symptoms and determined bacterial growth upon dip-inoculation of *N. benthamiana* plants (10^6^ cfu/ml; **Figure 4B**). Compared to *Pss* expressing *Pph*HopQ1 alone (**Figure 1A**), *Pss* expressing both *Pph*HopQ1 and *Pph*HopR1 induced strong disease symptoms and multiplied to high levels (**Figure 4B**). The operon with *PphhopQ1* and *PtohopR1* rendered *Pss* less virulent, similar to bacteria expressing *Pph*HopQ1 alone (**Figure 1A**). Inoculation with *Pss* expressing *Pto*HopQ1 led to blight disease symptom development no matter which HopR1 variant was co-expressed. In both *Pto*HopQ1 combinations, however, the macroscopic symptoms were less pronounced than upon infection with bacteria expressing *Pph*HopQ1 along with *Pph*HopR1. As bacterial titers did not perfectly reflect the disease symptoms induced by the different effector combinations, additional mechanisms are involved. Nevertheless, the findings are indicative of interplay between HopQ1 and HopR1 when delivered into plant cells by *Pss*.

To reduce additional effects of other bacterial effectors present in *Pss*, we used *Pto*DC3000D28E, a mutant strain of *P. syringae* pv. *tomato* DC3000 with 28 effector genes deleted (Cunnac et al., 2011), to specifically deliver various combinations of HopQ1 and HopR1. Importantly, this strain expresses HopAD1, that along with HopQ1 is required to trigger HR and as a consequence of single deletion of HopQ1 or HopAD1 *Pto*DC3000 gains virulence toward *N. benthamiana* (Wei et al., 2015). The transformed *Pto*DC3000D28E strains were infiltrated into leaves of *N. tabacum* plants to address whether they differ in their ability to induce hypersensitive cell death. None of the HopR1 variants triggered HR (**Figure 4C**), suggesting that HopR1 is not recognized in tobacco. In contrast, both HopQ1 variants expressed separately induced HR whereas the presence of HopR1 from the same *P. syringae* strain completely abolished this response. Interestingly, both combinations of HopQ1 and HopR1 derived from two different strains elicited HR. Furthermore, this response was stronger than triggered by HopQ1 variants alone. The fact that HopR1 expressed by bacteria infiltrated at very high inoculum (6 × 10^8^ CFU/ml) into *N. benthamiana* leaves triggers HR (Wei et al., 2018) suggests a possibility that HopR1 evokes cell death response also in *N. tabacum* and, thus, observed enhancement of tissue collapse (**Figure 4C**) would be due to synergistic/additive effect of HopQ1 and HopR1 action. The similarity between *Pph*HopR1 and *Pto*HopR1 is 86%, whereas it is 98% between *Pph*HopQ1 and *Pto*HopQ1. Collectively, these data suggest that the effector pairs co-evolved within a single strain and due to evolutionary diversification fail to co-operate when transferred individually from one strain to the other. This resembles a phenomenon described by Wei et al. (2015) when members of HopAB family displayed various abilities to suppress HopAD1 dependent cell death.

**FIGURE 5:**
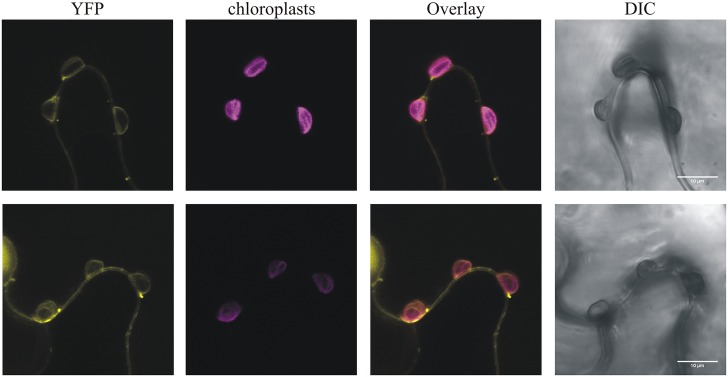
*Pph*HopR1 **(upper)** and *Pto*HopR1 **(lower)** transiently expressed in *N. benthamiana* localize to the cytoplasm and chloroplasts. Leaves were infiltrated with *Agrobacterium tumefaciens* strains carrying constructs encoding the HopR1 variants fused to eYFP. The images were recorded by confocal microscopy 72 h after agroinfiltration. DIC, differential interference contrast; bars = 10 μm.

This strain-specificity of HopR1 in blocking HR mediated by HopQ1 suggests that both effectors directly interact rather that HopR1 interfering with the signaling pathway initiated upon HopQ1 recognition. This model is, unfortunately, not consistent with the previous reports that HopQ1 is predominantly cytoplasmic (Giska et al., 2013; Li et al., 2013b), whereas HopR1 was shown to be imported into isolated chloroplasts (de Torres Zabala et al., 2015). However, HopR1 was detected both in chloroplasts and the cytoplasm when transiently expressed in *N. benthamiana* (**Figure 5**) and, thus, their association in the cytoplasm might still occur. The fact that in the native *Pto*DC3000 strain HopR1 is not able to block HopQ1-triggered cell death seems to be contradictory to our results. However, a similar case has been described for HopQ1 and HopI1 (Wei et al., 2018). Here, HopI1 was shown to block HopQ1 recognition in *N. benthamiana*. Although, *Pto*DC3000 that secretes both effectors is avirulent in this plant. In general, the (genetic) interactions between effectors are still poorly understood and, in this context, the mechanism of how HopR1 interferes with HopQ1 signaling requires further investigation.

Our data demonstrate that specific amino acid residues of *Pph*HopQ1 and *Pto*HopQ1 determine the disease outcome in *N. benthamiana* and *N. tabacum*. Sequence comparison showed that only six aa differ in the HopQ1 homologs studied. Two of these aa substantially affected HopQ1 properties. The presence of serine at position 87 and leucine at position 91 correlated with the susceptibility of the effector to the proteolytic cleavage within plant cells and debilitated effector recognition. Considering co-evolutionary adaptations, *P. syringae* would directly profit from HopQ1 cleavage, since HopQ1 recognition is avoided, even if it partially reduces its virulence properties in a susceptible host (**Figure 1**). The reduced virulence of the truncated form most likely results from the loss of interaction with 14-3-3 proteins in the host cell, since the HopQ1 N-terminus carries a canonical 14-3-3 binding site (RSX*p*SXP; *p*S indicates phosphoserine) that is important for proper effector localization and stability (Giska et al., 2013; Li et al., 2013b). From the “plant’s perspective,” cleavage would block the function of a single effector but would reduce sensing of the bacteria and, thereby, lead to disease development. Thus, we hypothesize that simultaneous maintenance of HopQ1 in the intact and truncated forms reflects a “calculated risk strategy” of *P. syringae*. In a susceptible plant, the virulence properties of the intact form sustain disease and the slightly reduced virulence properties of the truncated form still support bacterial proliferation. In a resistant plant, the N-terminally truncated form avoids recognition (Li et al., 2013a) and mediates suppression of a proper defense response. However, we cannot exclude that the cleavage of HopQ1 had been primarily a manifestation of the plant response that was later “corrupted” by *Pseudomonas*.

It was previously inferred from multilocus sequence typing that *P. syringae* pv. *tabaci* eliminated the sequence encoding HopQ1 from its genome to avoid detection (Ferrante et al., 2009). Our data suggest that besides this known mechanism, *P. syringae* may have evolved other strategies to prevent recognition. HopQ1 from *P. syringae* pv. *phaseolicola* (*Pph*HopQ1) co-evolved with HopR1 that masks its presence, pointing to tight co-adaptation in *P. syringae* pv. *phaseolicola*. In contrast, HopR1 from pathovar *tomato* was not able to block HR triggered by *Pph*HopQ1. Interestingly, *N. benthamiana* plants inoculated with *Pss* expressing *Pto*HopQ1 displayed a different phenotype than those plants inoculated with the strain expressing *Pph*HopQ1 (**Figure 1**). While introduction of Pph*hopQ1* rendered bacteria less virulent, Pto*hopQ1* compromised virulence of *Pss* to a lesser extent (**Figure 1**) which we hypothesize is linked to cleavage of *Pto*HopQ1 (**Figure 3**). Collectively, previous reports and our current results suggest that in order to avoid recognition of HopQ1, *P. syringae* evolved three different strategies that rely on (i) loss of the effector encoding sequences from its genome (Ferrante et al., 2009), (ii) partial susceptibility of the effector variants to proteolytic cleavage, and (iii) masking of the effector recognition by the co-adapted HopR1.

## Author Contributions

RH, LE-L, JL, JH, and MK conceived and designed the experiments. PZ, AD, RH, LE-L, MB, MG-B, IB-F, KM, JK, and MP performed the experiments. PZ, AD, RH, LE-L, MG-B, WR, JP, JL, JH, and MK analyzed the data. MK, LE-L, and JL wrote the paper.

## Conflict of Interest Statement

The authors declare that the research was conducted in the absence of any commercial or financial relationships that could be construed as a potential conflict of interest.

## Footnotes

1 http://string-db.org/
