# Supplementary material for: Two Strategies of Pseudomonas syringae to Avoid Recognition of the HopQ1 Effector in Nicotiana Species
Source: Front Plant Sci. 2018 Jul 10;9:978. doi: 10.3389/fpls.2018.00978 (PMC6048448; doi:10.3389/fpls.2018.00978)
Supplement: Supplementary file 1 [file Image_1.PDF]

*Supplementary Material*

# A Two strategies of *Pseudomonas syringae* to avoid recognition of the HopQ1 effector in *Nicotiana* species

**Patrycja Zembek[1]; Aleksandra Danilecka[1]; Rafał Hoser[1]; Lennart Eschen-Lippold[2]; Marta Benicka[1]; Marta Grech-Baran[1]; Wojciech Rymaszewski[1]; Izabela Barymow-Filoniuk[1]; Karolina Morgiewicz[1]; Jakub Kwiatkowski[1]; Marcin Piechocki[1]; Jaroslaw Poznanski[1]; Justin Lee[2]; Jacek Hennig[1]; Magdalena Krzymowska[1]***

*** Correspondence:** Magdalena Krzymowska: krzyma@ibb.waw.pl

**Supplementary Table 1. List of the strains used in this study.**

| Strain | Plasmid | Origin |
|---|---|---|
| ***Pseudomonas syringae* pv. *syringae* B728a** | | (Vinatzer et al., 2006) |
| ***Pseudomonas syringae* pv. *syringae* B728a** | pBBR1MCS-2-tac-mCherry | (Giska et al., 2013) |
| ***Pseudomonas syringae* pv. *syringae* B728a** | pBBR1MCS-2-tac-PtoHopQ1-6xHis-PtoHopR1-FLAG | This study |
| ***Pseudomonas syringae* pv. *syringae* B728a** | pBBR1MCS-2-tac-PtoHopQ1-6xHis-PphHopR1-FLAG | This study |
| ***Pseudomonas syringae* pv. *syringae* B728a** | pBBR1MCS-2-tac-PphHopQ1-6xHis-PtoHopR1-FLAG | This study |
| ***Pseudomonas syringae* pv. *syringae* B728a** | pBBR1MCS-2-tac-PphHopQ1-6xHis-PphHopR1-FLAG | This study |
| ***Pseudomonas syringae* pv. *syringae* B728a** | pBBR1MCS-2-tac-PtoHopQ1-6xHis | This study |
| ***Pseudomonas syringae* pv. *syringae* B728a** | pBBR1MCS-2-tac-PphHopQ1-6xHis | (Giska et al., 2013) |
| ***Pseudomonas syringae* pv. *syringae* B728a** | pBBR1MCS-2-tac-PtoHopQ1-S87L_L91R-6xHis | This study |
| ***Pseudomonas syringae* pv. *tomato* DC3000** | | (Buell et al., 2003) |
| ***Pseudomonas syringae* pv. *tomato* DC3000 D28E** | | (Cunnac et al., 2011) |
| ***Pseudomonas syringae* pv. *tomato* DC3000 D28E** | pBBR1MCS-2-tac-mCherry | (Giska et al., 2013) |
| ***Pseudomonas syringae* pv. *tomato* DC3000 D28E** | pBBR1MCS-2-tac-PtoHopQ1-6xHis-PtoHopR1-FLAG | This study |
| ***Pseudomonas syringae* pv. *tomato* DC3000** | pBBR1MCS-2-tac-PtoHopQ1-6xHis- | This study |

| | | |
|---|---|---|
| **D28E** | PphHopR1-FLAG | |
| ***Pseudomonas syringae* pv. *tomato* DC3000 D28E** | pBBR1MCS-2-tac-PphHopQ1-6xHis-PtoHopR1-FLAG | This study |
| ***Pseudomonas syringae* pv. *tomato* DC3000 D28E** | pBBR1MCS-2-tac-PphHopQ1-6xHis-PphHopR1-FLAG | This study |
| ***Pseudomonas syringae* pv. *phaseolicola* 1448a** | | (Joardar et al., 2005) |
| ***Agrobacterium tumefaciens* GV3101** | pGWB-441-PtoHopR1 | This study |
| ***Agrobacterium tumefaciens* GV3101** | pGWB-441-PphHopR1 | This study |

**Buell, C.R., Joardar, V., Lindeberg, M., Selengut, J., Paulsen, I.T., Gwinn, M.L., et al. (2003). The complete genome sequence of the Arabidopsis and tomato pathogen *Pseudomonas syringae* pv. *tomato* DC3000. *Proceedings of the National Academy of Sciences of the United States of America* 100(18), 10181-10186. doi: 10.1073/pnas.1731982100.**

**Cunnac, S., Chakravarthy, S., Kvitko, B.H., Russell, A.B., Martin, G.B., and Collmer, A. (2011). Genetic disassembly and combinatorial reassembly identify a minimal functional repertoire of type III effectors in *Pseudomonas syringae*. *Proceedings of the National Academy of Sciences of the United States of America* 108(7), 2975-2980.**

**Giska, F., Lichocka, M., Piechocki, M., Dadlez, M., Schmelzer, E., Hennig, J., et al. (2013). Phosphorylation of HopQ1, a Type III Effector from *Pseudomonas syringae*, Creates a Binding Site for Host 14-3-3 Proteins. *Plant physiology* 161(4), 2049-2061. doi: 10.1104/pp.112.209023.**

**Joardar, V., Lindeberg, M., Jackson, R.W., Selengut, J., Dodson, R., Brinkac, L.M., et al. (2005). Whole-genome sequence analysis of *Pseudomonas syringae* pv. *phaseolicola* 1448A reveals divergence among pathovars in genes involved in virulence and transposition. *Journal of bacteriology* 187(18), 6488-6498. doi: 10.1128/JB.187.18.6488-6498.2005.**

**Vinatzer, B.A., Teitzel, G.M., Lee, M.W., Jelenska, J., Hotton, S., Fairfax, K., et al. (2006). The type III effector repertoire of *Pseudomonas syringae* pv. *syringae* B728a and its role in survival and disease on host and non-host plants. *Molecular microbiology* 62(1), 26-44. doi: 10.1111/j.1365-2958.2006.05350.x.**

**Supplementary Table 2. List of the primers used in this study.**

| Primer | Primer sequence (5’→3’) | Description |
|---|---|---|
| **F-BamHI-HopQ1**<br><br>**R-HopQ1-XhoI** | **GGATCC**ATGCATCGTCCTATCACCGC<br><br>TACAT**CTCGAG**TCAATCTGGGGCTACCGTCGAC TGG | Cloning of *PphhopQ1* or *PtohopQ1-1* genes into pBBRMCS-X vectors using BamHI and XhoI restriction sites (Giska et al., 2013) |
| **F-HopQ1-6xHIS**<br><br>**R-HopQ1-6xHIS** | TCGACGGTAGCCCCAGAT**CTCGAG**CACCACCA CCACCACCACTGAGGATCCGGTAC<br><br>CGGATCCTCAGTGGTGGTGGTGGTGGTG**CTCG AG**ATCTGGGGCTACCG | *Addition of* the 6×*His encoding sequence* to the 3’ end of the *PphhopQ1* or *PtohopQ1-1* gene sequence cloned in pBBRMCS-X vectors sites (Giska F. *et al.*, Plant Physiol. 2013) |
| **F-PphHopQ1_L19S**<br><br>**R-PphHopQ1_L19S** | TCCTAGATCAGTCAAAACAAATATCACGTACCC C<br><br>TGAGACGTGAGGTGGTATGGC | Introduction of the point mutation (L19S) into the *PphhopQ1* gene sequence |
| **F-PphHopQ1_V31A**<br><br>**R-PphHopQ1_V31A** | ATCGGAAAGTAGCGCGCAATCAGCAC<br><br>GGGGTACGTGATATTTGTTTTAACTG | Introduction of the point mutation (V31A) into the sequence of *PphhopQ1* |
| **F-PphHopQ1_S72A**<br><br>**R-PphHopQ1_S72A** | ACAAGTGGGAGCCTGTAACGCTCATCTGAC<br><br>GCAAGCATCGTGCGCTGTG | Introduction of the point mutation (S72A) into *PphhopQ1*$_{L19S}$ gene to obtain double mutant *PphhopQ1*$_{L19S\_S72A}$ |
| **F-PphHopQ1_G154E**<br><br>**PphHopQ1_G154E-rev** | CAACAAGCTTGAGTTGCATGACGTGCATGTG<br><br>AACACGCCTTTGGCCATCTCG | Introduction of the point mutation (G154E) into *PphhopQ1*$_{L19S}$ sequence to obtain double mutant *PphhopQ1*$_{L19S\_G154E}$ |
| **F-PphHopQ1_L87S_R91L**<br><br>**R-PphHopQ1_L87S_R91L** | AGATCACACAAGCCCCTGTTACCTAAG<br><br>TAACAGGGGCTTGTGTGATCTCAGTTC | Introduction of the point mutation (L87S and R91L) into *PphhopQ1* sequence |
| **F-PtoHopQ1-1_S87L_L91R**<br><br>**R-PtoHopQ1-1_S87L_L91R** | CGAACTGAGACTACACAAGCCCAGATTACCTA AG<br><br>TTGATGGCCATGTTTTCATCTG | Introduction of the mutations (S87L and L91R) into *PtohopQ1-1* sequence |

| | | |
|---|---|---|
| **F-PphHopR1-pENTR**<br><br>**R-PphHopR1-pENTR** | CACCATGGTAAAGGTTACCTCTTCC<br><br>CTGCGGCATCTTATCGAGTTC | Cloning of *PphhopR1* gene into pENTR D-TOPO vector |
| **F-PtoHopR1-pENTR**<br><br>**R-PtoHopR1-pENTR** | CACCATGGTCAAGGTTACCTCTTCC<br><br>CACGTTATCGAGTTCGCCCC | Cloning of *PtohopR1* gene into pENTR D-TOPO vector |
| ***Cloning of PphhopR1 or PtoHopR1* genes using KpnI restriction sites to construct synthetic operons with *PphhopQ1* or *PtohopQ1-1* genes in pBBRMCS-X** | | |
| **F-KpnI-RBS-PphR1**<br><br>**R- PphR1-FLAG-KpnI** | **GGTACC**TTAACTTTAAGAAGGAGCCCTTCACCATGGTAAAGGTTAC<br><br>**GGTACC**TTACTTGTCATCGTCATCCTTGTAGTCCGCCGCCTGCGGCATCTTATCGAGTTC | Addition of the ribosome binding site (RBS=TTAACTTTAAGAAGGAGCCCTT) encoding sequence in front of the *PtohopR1* DNA sequence and FLAG *tag encoding sequence to* the 3' end of the *PphHopR1* gene<br><br>The cloned region RBS-*PphhopR1*-FLAG was flanked by KpnI restriction sites allowing insertion into the pBBRMCS-X containing *PphhopQ1 or PtohopQ1-1* gene |
| **F-PtoR1-pENTR-attL2**<br><br>**R- PtoR1-FLAG-KpnI** | AAGGGTGGGCGCGCCGAC<br><br>**GGTACC**TTACTTGTCATCGTCATCCTTGTAGTCCGCCGCCACGTTATCGAGTTCGCCCC | Addition of the FLAG tag encoding sequence and KpnI restriction enzyme sites to the 3' end of the *PtoHopR1* sequence |
| **F-pENTR-RBS-PtoHopR1**<br><br>**R-PtoR1-pENTR-attL1** | CACC**GGTACC**TTAACTTTAAGAAGGAGCCCTTCACCATGGTCAAGGTTAC<br><br>GGAGCCTGCTTTTTTGTACAAAG | Addition of the KpnI restriction site and RBS en*coding sequences* to the 5' end of the *PtoHopR1 gene* sequence containing already FLAG tag and KpnI resrtriction site encoding sequences at the 3' end of the *PtoHopR1* |

*Bolded regions in the primers represent the cleavage sites for the restriction enzymes mentioned in the description column

**Underlined regions in the primers represent the tag sequence mentioned in the description column

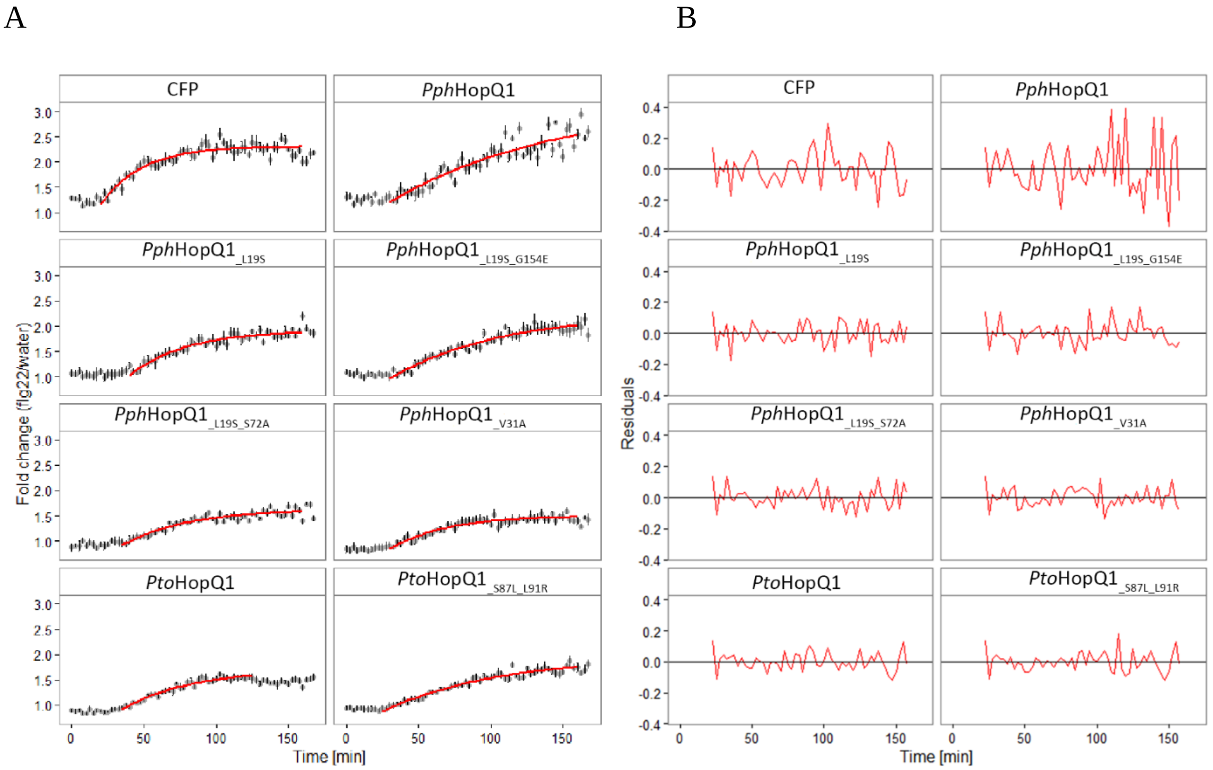


**Supplementary Figure 1** Fold changes (FCs) from *pNHL10-LUC* assays performed on Arabidopsis protoplasts. (A) Each point corresponds to a flg22/water ratio at a given time point. Curve fitting was done using an exponential function: $FC = F0 + (Fmax - F0) \times (1 - e^{\left(-\frac{t}{\tau}\right)})$, where $\tau$ is the time constant proportional to the curvature of the slope, *F0* is the initial fluorescence fold change, *Fmax* is maximum fluorescence fold change, and *t* is time (min) at a given point. Time intervals for curve fitting were chosen individually for each protein variant. (B) Residual profiles obtained after curve fitting.

**Supplementary Figure 2 A putative subtilisin cleavage site is present in *Pto*HopQ1.** ELM analyses of the HopQ1 variants were performed under default conditions (Dinkel et al. , 2016).

**Dinkel, H., Van Roey, K., Michael, S., Kumar, M., Uyar, B., Altenberg, B., et al. (2016). ELM 2016--data update and new functionality of the eukaryotic linear motif resource. *Nucleic acids research* 44(D1), D294-300. doi: 10.1093/nar/gkv1291.**

## Summary for sequence 'PphHopQ1'.

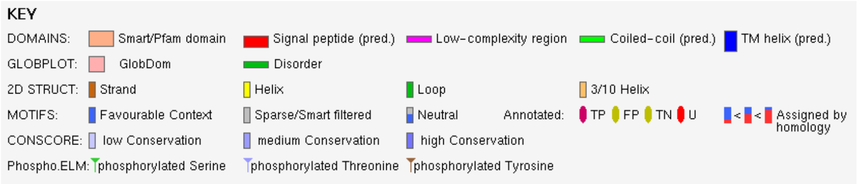


(Mouseover the matches for more details )

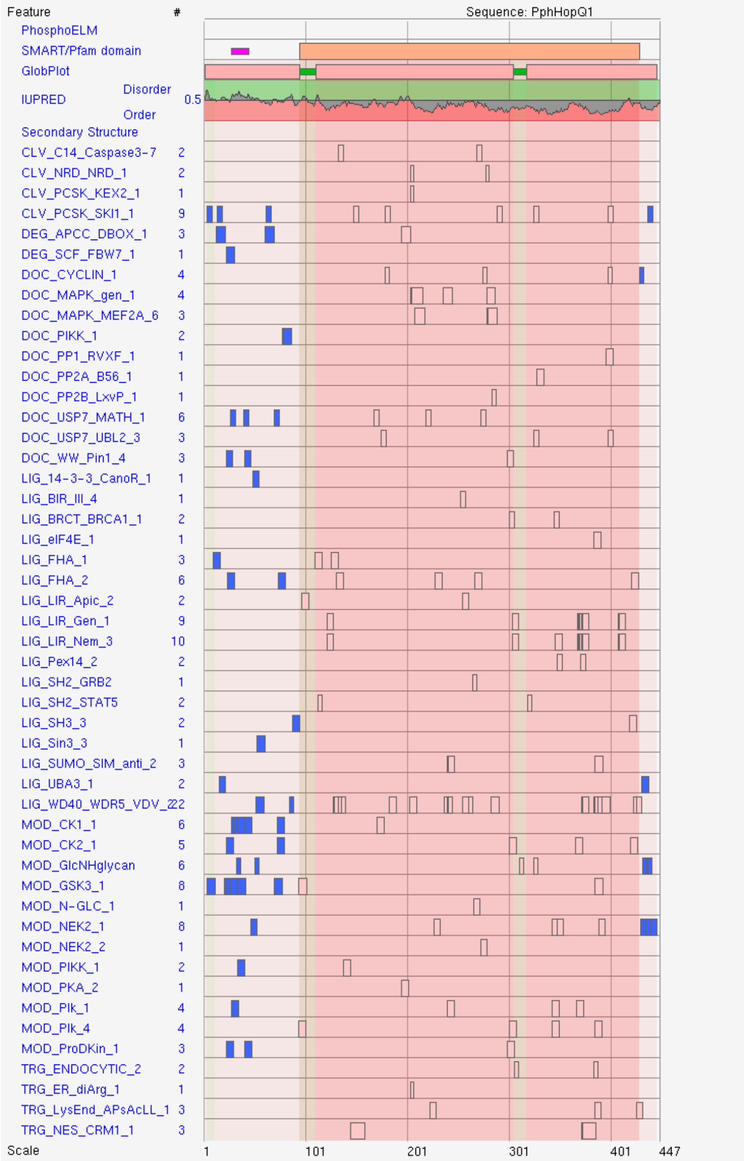


Either not enough data available to calculate a sequence alignment or the calculations haven't finished yet (in which case you can try to reload the page)...

## Filtering summary

**No user supplied cellular location.**
**User supplied taxon: root**

(An ELM is listed as filtered when all its matching instances have been filtered out.)

| | | Elms | Instances |
|---|---|---|---|
| **FILTERED BY:** | Species | 0 | 0 |
| | Cellular location (counts only those ELMs not already excluded by species.) | 0 | 0 |
| | Structural score (below medium threshold score) | 0 | 0 |
| | Smart (in a domain and no structural filter info available) | 27 | 124 |
| **TOTAL FILTERED:** | | 27 | 124 |
| **RETAINED BY:** | Smart (outside domain and no structural filter info available) | 22 | 50 |
| | Structural score (at or above medium threshold score) | 0 | 0 |
| **TOTAL RETAINED:** | | 22 | 50 |
| **TOTAL** | all found (before filtering) | 49 | 174 |

Query sequence:
>PphHopQ1
MHRPITAGHTTSRLILDQLKQISRTPSESSVQSALSQQASMSSPVLERSKSAPALLTAAQ
RTMLAQVGACNSHLTSDENMAINELRLHKPRLPKDTWFFTDPNKDPDDVVTYTLGKQLQA
EGFVHITDVVATLGDAEVRSQRAEMAKGVFNKLGLHDVHVSRGRDYAMNSLQSKEHAKFL
LEGHALRAGPGEIHRDSLQDMSRRLARAPHGVGIVVIAGMSDINALITTCPDMVRERVDD
ITIMGGVEPLKDADGFVQPDARAYNNATDMDAARSLYRKAQELGIPLRIVTKEAAYKTAV
SPSFYEGIAGSGHPVGHYLRDVQKSALKGLWEGIQAGLLPGLDDSWFFRTFMPNAQIEAA
QLDKNKESSFEDIWPKVTKLNLYDPLTLLASVPGAAKLLFKPKAIHTEGFGVVEQVGPDD
VTHPEKAKLLMSALAKSALVQSTVAPD

## Globular domains/ TM domains and signal peptide detected by the SMART server

| Domain | Start | End |
|---|---|---|
| Pfam:IU_nuc_hydro | 94 | 427 |

## Results of ELM motif search after globular domain filtering, structural filtering and context filtering.

### Matches falling inside globular protein domains are excluded from this list unless having an acceptable structural score (if the structural filter (BETA version) is applicable). If the structural filter (BETA version)is applicable it is possible to view these structures with Jmol

| Elm Name | Instances (Matched Sequence) | Positions | View in Jmol | Elm Description | Cell Compartment | Pattern | PHI-Blast Instance Mapping | Structural Filter Info | Probability |
|---|---|---|---|---|---|---|---|---|---|
| CLV_PCSK_SKI1_1 | RPITA<br>RLILD<br>RTMLA<br>KSALV | 3-7 [A]<br>13-17 [A]<br>61-65 [A]<br>436-440 [A] | -<br>-<br>-<br>- | Subtilisin/kexin isozyme-1 (SKI1) cleavage site ([RK]-X-[hydrophobic]-[LTKF]-\|-X). | extracellular, Golgi apparatus, endoplasmic reticulum lumen, endoplasmic reticulum | [RK].[AILMFV][LTKF]. | - | - | 6.821e-03 |
| DEG_APCC_DBOX_1 | SRLILDQLK<br>QRTMLAQVG | 12-20 [A]<br>60-68 [A] | -<br>- | An RxxL-based motif that binds to the Cdh1 and Cdc20 components of APC/C thereby targeting the protein for destruction in a cell cycle dependent manner | nucleus, cytosol | .R..L..[LIVM]. | - | - | 7.677e-04 |
| DEG_SCF_FBW7_1 | ISRTPSES | 22-29 [A] | - | The TPxxS phospho-dependent degron binds the FBW7 F box proteins of the SCF (Skp1_Cullin-Fbox) complex. | nucleus, cytosol | [LIVMP].{0,2}(T)P..([ST]) | - | - | 7.138e-04 |
| DOC_CYCLIN_1 | KLLM | 428-431 [A] | - | Substrate recognition site that interacts with cyclin and thereby increases phosphorylation by cyclin/cdk complexes. Predicted proteins should have a CDK phosphorylation site. Also used by cyclin/cdk inhibitors. | nucleus, cytosol | [RK].L.{0,1}[FYLIVMP] | - | - | 5.324e-03 |
| DOC_PIKK_1 | DENMAINEL<br>ENMAINEL | 77-85 [A]<br>78-85 [A] | -<br>- | DOC_PIKK_1 motif is located in the C terminus of Nbs1 and its homologues and interacts with PIKK family members. | nucleus | [DEN][DEN].{2,3}[ILMVA][DEN][DEN]L | - | - | 3.129e-05 |
| DOC_USP7_MATH_1 | PSESS<br>ASMSS<br>ACNSH | 26-30 [A]<br>39-43 [A]<br>69-73 [A] | -<br>-<br>- | The USP7 MATH domain binding motif variant based on the MDM2 and p53 interactions. | nucleus | [PA][^P][^FYWIL]S[^P] | - | - | 1.239e-02 |
| DOC_WW_Pin1_4 | ISRTPS<br>SMSSPV | 22-27 [A]<br>40-45 [A] | -<br>- | The Class IV WW domain interaction motif is recognised primarily by the Pin1 phosphorylation-dependent prolyl isomerase. | cytosol, nucleus | ...([ST])P. | - | - | 1.543e-02 |
| LIG_14-3-3_CanoR_1 | RSKSAP | 48-53 [A] | - | Canonical Arg-containing phospho-motif mediating a strong interaction with 14-3-3 proteins. | cytosol, internal side of plasma membrane, nucleus | R[^DE]{0,2}[^DEPG]([ST])(([FWYLMV].)\|([^PRIKGN]P)\|([^PRIKGN].{2,4}[VILMFWYP])) | - | - | 4.477e-03 |

| | | | | | | | | | |
|---|---|---|---|---|---|---|---|---|---|
| LIG_FHA_1 | HTTSRLI | 9-15 [A] | - | Phosphothreonine motif binding a subset of FHA domains that show a preference for a large aliphatic amino acid at the pT+3 position. | nucleus | ..(T)..[ILV]. | - | - | 8.662e-03 |
| LIG_FHA_2 | SRTPSES<br>HLTSDEN | 23-29 [A]<br>73-79 [A] | -<br>- | Phosphothreonine motif binding a subset of FHA domains that have a preference for an acidic amino acid at the pT+3 position. | nucleus,<br>Replication fork | ..(T)..[DE]. | - | - | 8.286e-03 |
| LIG_SH3_3 | LHKPRLP | 87-93 [A] | - | This is the motif recognized by those SH3 domains with a non-canonical class I recognition specificity | plasma membrane,<br>focal adhesion,<br>cytosol | ...[PV]..P | - | - | 1.317e-02 |
| LIG_Sin3_3 | APALLTAA | 52-59 [A] | - | Motif interacts with PAH2 domain in the Sin3 scaffold protein (not mad or sp-1 like). | nucleus | [FA].[LA][LV][LVI]..[AM] | - | - | 2.197e-05 |
| LIG_UBA3_1 | ILDQLK<br>LMSALAK | 15-20 [A]<br>430-436 [A] | -<br>- | UBA3 adenylation domain binding motif variant based on the UBE2M and UBE2F interactions. | nucleus | [ILM][ILMF].{1,2}[ILM].{0,4}K | - | - | 1.196e-03 |
| LIG_WD40_WDR5_VDV_2 | SAPALLTA<br>ELRL | 51-58 [A]<br>84-87 [A] | -<br>- | Fungi-specific variant of the WDR5-binding motif that binds to a cleft between blades 5 and 6 of the WD40 repeat domain of WDR5, opposite of the Win motif-binding site, to mediate assembly of histone modification complexes. | nucleus,<br>histone methyltransferase complex | [EDSTY].{0,4}[VIPLA][TSDEKR][ILVA] | - | - | 4.678e-02 |
| MOD_CK1_1 | SESSVQS<br>SVQSALS<br>SALSQQA<br>SMSSPVL<br>SHLTSDE | 27-33 [A]<br>30-36 [A]<br>33-39 [A]<br>40-46 [A]<br>72-78 [A] | -<br>-<br>-<br>-<br>- | CK1 phosphorylation site | nucleus,<br>cytosol | S..([ST])... | - | - | 1.704e-02 |
| MOD_CK2_1 | ISRTPSE<br>SHLTSDE | 22-28 [A]<br>72-78 [A] | -<br>- | CK2 phosphorylation site | nucleus,<br>protein kinase CK2 complex,<br>cytosol | ...([ST])..E | - | - | 1.457e-02 |
| MOD_GlcNHglycan | QSAL<br>KSAP<br>MSAL<br>KSAL | 32-35 [A]<br>50-53 [A]<br>431-434 [A]<br>436-439 [A] | -<br>-<br>-<br>- | Glycosaminoglycan attachment site | extracellular,<br>Golgi apparatus | [ED]{0,3}.(S)[GA]. | - | - | 1.792e-02 |
| MOD_GSK3_1 | RPITAGHT<br>KQISRTPS<br>ISRTPSES<br>PSESSVQS<br>SALSQQAS<br>ACNSHLTS | 3-10 [A]<br>20-27 [A]<br>22-29 [A]<br>26-33 [A]<br>33-40 [A]<br>69-76 [A] | -<br>-<br>-<br>-<br>-<br>- | GSK3 phosphorylation recognition site | nucleus,<br>cytosol | ...([ST])...[ST] | - | - | 2.679e-02 |
| MOD_NEK2_1 | LERSKS<br>LLMSAL<br>LAKSAL<br>LVQSTV | 46-51 [A]<br>429-434 [A]<br>434-439 [A]<br>439-444 [A] | -<br>-<br>-<br>- | NEK2 phosphorylation motif with preferred Phe, Leu or Met in the -3 position to compensate for less favorable residues in the +1 and +2 position. | centrosome,<br>Ndc80 complex,<br>condensed nuclear chromosome outer kinetochore,<br>cytosol,<br>nucleus | [FLM][^P][^P]([ST])[^DEP][^DE] | - | - | 9.798e-03 |
| MOD_PIKK_1 | SALSQQA | 33-39 [A] | - | (ST)Q motif which is phosphorylated by PIKK family members. | nucleus | ...([ST])Q.. | - | - | 9.230e-03 |
| MOD_Plk_1 | SESSVQS | 27-33 [A] | - | Ser/Thr residue phosphorylated by the Plk1 kinase | centralspindlin complex,<br>nucleus,<br>spindle,<br>gamma-tubulin complex,<br>midbody,<br>cytosol,<br>kinetochore,<br>spindle midzone,<br>nuclear condensin complex, | .[DNE][^PG][ST](([FYILMVW]..)\|([^PEDGKN][FWYLIVM]).) | - | - | 7.674e-03 |

| | | | | | | | | | |
|---|---|---|---|---|---|---|---|---|---|
| | | | | | cleavage furrow, nucleoplasm, microtubule organizing center | | | | |
| MOD_ProDKin_1 | ISRTPSE SMSSPVL | 22-28 [A] 40-46 [A] | - - | Proline-Directed Kinase (e.g. MAPK) phosphorylation site in higher eukaryotes. | nucleus, cytosol | ...([ST])P.. | - | - | 1.543e-02 |

## List of excluded ELMs falling inside SMART/PFAM domains and/or scoring poorly with the structural filter (if applicable).

**Matches in this list are only likely to be of interest if they are in accessible surface-exposed loops. Motif matches buried in stably folded cores of globular domains are not plausible candidates.**
**If the structural filter (BETA version) is applicable it is possible to view these structures with Jmol. For more info consult the PDB structure entry used for structure filtering or the SMART or PFAM entries for useful links to solved 3D structures.**

| Elm Name | Positions | View in Jmol | Elm Description | Cell Compartment | Pattern | PHI-Blast Instance Mapping | Structural Filter Info | Probability |
|---|---|---|---|---|---|---|---|---|
| CLV_C14_Caspase3-7 | 132-136 [A] 268-272 [A] | - - | Caspase-3 and Caspase-7 cleavage site. | nucleus, cytosol | [DSTE][^P][^DEWHFYC]D[GSAN] | - | - | 3.094e-03 |
| CLV_NRD_NRD_1 | 203-205 [A] 277-279 [A] | - - | N-Arg dibasic convertase (NRD/Nardilysin) cleavage site (X-\|-R-K or R-\|-R-X). | extracellular, Golgi apparatus, cell surface | (.RK)\|(RR[^KR]) | - | - | 7.465e-03 |
| CLV_PCSK_KEX2_1 | 203-205 [A] | - | Yeast kexin 2 cleavage site (K-R-\|-X or R-R-\|-X). | extracellular, Golgi apparatus | [KR]R. | - | - | 7.973e-03 |
| CLV_PCSK_SKI1_1 | 147-151 [A] 178-182 [A] 288-292 [A] 324-328 [A] 397-401 [A] | - - - - - | Subtilisin/kexin isozyme-1 (SKI1) cleavage site ([RK]-X-[hydrophobic]-[LTKF]-\|-X). | extracellular, Golgi apparatus, endoplasmic reticulum lumen, endoplasmic reticulum | [RK].[AILMFV][LTKF]. | - | - | 6.821e-03 |
| DEG_APCC_DBOX_1 | 194-202 [A] | - | An RxxL-based motif that binds to the Cdh1 and Cdc20 components of APC/C thereby targeting the protein for destruction in a cell cycle dependent manner | nucleus, cytosol | .R..L..[LIVM]. | - | - | 7.677e-04 |
| DOC_CYCLIN_1 | 178-181 [A] 274-277 [A] 397-400 [A] | - - - | Substrate recognition site that interacts with cyclin and thereby increases phosphorylation by cyclin/cdk complexes. Predicted proteins should have a CDK phosphorylation site. Also used by cyclin/cdk inhibitors. | nucleus, cytosol | [RK].L.{0,1}[FYLIVMP] | - | - | 5.324e-03 |
| DOC_MAPK_gen_1 | 203-214 [A] 204-214 [A] 235-243 [A] 278-285 [A] | - - - - | MAPK interacting molecules (e.g. MAPKKs, substrates, phosphatases) carry docking motif that help to regulate specific interaction in the MAPK cascade. The classic motif approximates (R/K)xxxx#x# where # is a hydrophobic residue. | nucleus, cytosol | [KR]{0,2}[KR].{0,2}[KR].{2,4}[ILVM].[ILVF] | - | - | 4.324e-03 |
| DOC_MAPK_MEF2A_6 | 207-216 [A] | - | A kinase docking motif | cytosol, Transcription | [RK].{2,4}[LIVMP].[LIV].[LIVMF] | - | - | 2.584e-03 |

| | | | | | | | | |
|---|---|---|---|---|---|---|---|---|
| | 278-287 [A] 279-287 [A] | - - | that mediates interaction towards the ERK1/2 and p38 subfamilies of MAP kinases. | factor complex, nucleus | | | | |
| DOC_PP1_RVXF_1 | 395-401 [A] | - | Protein phosphatase 1 catalytic subunit (PP1c) interacting motif binds targeting proteins that dock to the substrate for dephosphorylation. The motif defined is [RK]{0,1}[VI][^P][FW]. | nucleus, protein phosphatase type 1 complex, cytosol | ..[RK].{0,1}[VIL][^P][FW]. | - | - | 8.301e-04 |
| DOC_PP2A_B56_1 | 327-333 [A] | - | Docking site required for the regulatory subunit B56 of PP2A for protein dephosphorylation. | nucleus, kinetochore, chromosome, centromeric region, cytosol | ([LMFYWIC]..I.E)\|(L..[IVLWC].E). | - | - | 1.458e-03 |
| DOC_PP2B_LxvP_1 | 283-286 [A] | - | Docking motif in calcineurin substrates that binds at the interface of the catalytic CNA and regulatory CNB subunits. | cytosol, calcineurin complex, nucleus | L.[LIVAPM]P | - | - | 2.296e-03 |
| DOC_USP7_MATH_1 | 167-171 [A] 218-222 [A] 272-276 [A] | - - - | The USP7 MATH domain binding motif variant based on the MDM2 and p53 interactions. | nucleus | [PA][^P][^FYWIL]S[^P] | - | - | 1.239e-02 |
| DOC_USP7_UBL2_3 | 174-178 [A] 324-328 [A] 397-401 [A] | - - - | The USP7 CTD domain binding motif variant based on the ICP0 and DNMT1 interactions | nucleus | K...K | - | - | 3.742e-03 |
| DOC_WW_Pin1_4 | 298-303 [A] | - | The Class IV WW domain interaction motif is recognised primarily by the Pin1 phosphorylation-dependent prolyl isomerase. | cytosol, nucleus | ...([ST])P. | - | - | 1.543e-02 |
| LIG_BIR_III_4 | 252-256 [A] | - | These IBMs are found in the N-terminal regions of arthropodal caspase subunits where they mediate the inhibition of activated caspases by binding to conserved surface grooves on type III BIR domains of Inhibitor of Apoptosis Proteins (IAPs). | mitochondrion, cytosol | DA.G. | - | - | 2.754e-04 |
| LIG_BRCT_BRCA1_1 | 300-304 [A] 344-348 [A] | - - | Phosphopeptide motif which directly interacts with the BRCT (carboxy-terminal) domain of the Breast Cancer Gene BRCA1 with low affinity | nucleus, BRCA1-BARD1 complex | .(S)..F | - | - | 1.912e-03 |
| LIG_eIF4E_1 | 383-389 [A] | - | Motif binding to the dorsal surface of eIF4E. | cytosol | Y....L[VILMF] | - | - | 1.891e-04 |
| LIG_FHA_1 | 109-115 [A] 125-131 [A] | - - | Phosphothreonine motif binding a subset of FHA domains that show a preference for a large aliphatic amino acid at the pT+3 position. | nucleus | ..(T)..[ILV]. | - | - | 8.662e-03 |
| LIG_FHA_2 | 130-136 [A] 227-233 [A] 266-272 [A] 420-426 [A] | - - - - | Phosphothreonine motif binding a subset of FHA domains that have a preference for an acidic amino acid at the pT+3 position. | nucleus, Replication fork | ..(T)..[DE]. | - | - | 8.286e-03 |

| | | | | | | | | |
|---|---|---|---|---|---|---|---|---|
| LIG_LIR_Apic_2 | 96-102 [A] 254-259 [A] | - - | Apicomplexa specific variant of the canonical LIR motif that binds to Atg8 protein family members to mediate processes involved in autophagy. | cytosol, cytoplasmic side of late endosome membrane | [EDST].{0,2}[WFY]..P | - | - | 3.371e-03 |
| LIG_LIR_Gen_1 | 121-126 [A] 303-308 [A] 367-373 [A] 368-373 [A] 369-373 [A] 371-377 [A] 372-377 [A] 407-413 [A] 408-413 [A] | - - - - - - - - - | Canonical LIR motif that binds to Atg8 protein family members to mediate processes involved in autophagy. | cytosol, cytoplasmic side of late endosome membrane | [EDST].{0,2}[WFY]..[ILV] | - | - | 5.200e-03 |
| LIG_LIR_Nem_3 | 121-126 [A] 303-308 [A] 345-351 [A] 367-373 [A] 368-373 [A] 369-373 [A] 371-377 [A] 372-377 [A] 407-413 [A] 408-413 [A] | - - - - - - - - - - | Nematode-specific variant of the canonical LIR motif that binds to Atg8 protein family members to mediate processes involved in autophagy. | cytosol, cytoplasmic side of late endosome membrane | [EDST].{0,2}[WFY]..[ILVFY] | - | - | 6.362e-03 |
| LIG_Pex14_2 | 347-351 [A] 370-374 [A] | - - | Fxxx[WF] motifs are present in Pex19 and S. cerevisiae Pex5 cytosolic receptors that bind to peroxisomal membrane docking member, Pex14 | cytosol, peroxisome, glycosome | F...[WF] | - | - | 4.628e-04 |
| LIG_SH2_GRB2 | 264-267 [A] | - | GRB2-like Src Homology 2 (SH2) domains binding motif. | cytosol | (Y).N. | - | - | 4.787e-04 |
| LIG_SH2_STAT5 | 112-115 [A] 318-321 [A] | - - | STAT5 Src Homology 2 (SH2) domain binding motif. | cytosol | (Y)[VLTFIC].. | - | - | 3.296e-03 |
| LIG_SH3_3 | 418-424 [A] | - | This is the motif recognized by those SH3 domains with a non-canonical class I recognition specificity | plasma membrane, focal adhesion, cytosol | ...[PV]..P | - | - | 1.317e-02 |
| LIG_SUMO_SIM_anti_2 | 239-245 [A] 240-245 [A] 384-391 [A] | - - - | Motif for the antiparallel beta augmentation mode of non-covalent binding to SUMO protein. | nucleus, PML body, nuclear body | [DEST]{1,10}.{0,1}[VIL][DESTVILMA][VIL][VILM].[DEST]{0,5} | - | - | 2.349e-03 |
| LIG_WD40_WDR5_VDV_2 | 127-133 [A] 128-133 [A] 132-138 [A] 135-138 [A] 182-188 | - - - - - - - - | | nucleus, histone methyltransferase complex | [EDSTY].{0,4}[VIPLA][TSDEKR][ILVA] | - | - | 4.678e-02 |

| | | | | | | | | |
|---|---|---|---|---|---|---|---|---|
| | [A]<br>202-208<br>[A]<br>236-243<br>[A]<br>239-243<br>[A]<br>240-243<br>[A]<br>254-261<br>[A]<br>260-263<br>[A]<br>282-289<br>[A]<br>371-377<br>[A]<br>372-377<br>[A]<br>383-388<br>[A]<br>384-388<br>[A]<br>387-392<br>[A]<br>391-398<br>[A]<br>422-429<br>[A]<br>425-429<br>[A] | -<br>-<br>-<br>-<br>-<br>-<br>-<br>-<br>-<br>-<br>-<br>- | Fungi-specific variant of the WDR5-binding motif that binds to a cleft between blades 5 and 6 of the WD40 repeat domain of WDR5, opposite of the Win motif-binding site, to mediate assembly of histone modification complexes. | | | | | |
| MOD_CK1_1 | 170-176<br>[A] | - | CK1 phosphorylation site | nucleus, cytosol | S..([ST])... | - | - | 1.704e-02 |
| MOD_CK2_1 | 300-306<br>[A]<br>365-371<br>[A]<br>419-425<br>[A] | -<br>-<br>- | CK2 phosphorylation site | nucleus, protein kinase CK2 complex, cytosol | ...([ST])..E | - | - | 1.457e-02 |
| MOD_GlcNHglycan | 310-313<br>[A]<br>324-327<br>[A] | -<br>- | Glycosaminoglycan attachment site | extracellular, Golgi apparatus | [ED]{0,3}.(S)[GA]. | - | - | 1.792e-02 |
| MOD_GSK3_1 | 93-100<br>[A]<br>384-391<br>[A] | -<br>- | GSK3 phosphorylation recognition site | nucleus, cytosol | ...([ST])...[ST] | - | - | 2.679e-02 |
| MOD_N-GLC_1 | 265-270<br>[A] | - | Generic motif for N-glycosylation. It was shown that Trp, Asp, and Glu are uncommon before the Ser/Thr position. Efficient glycosylation usually occurs when ~60 residues or more separate the glycosylation acceptor site from the C-terminus. | extracellular, Golgi apparatus, endoplasmic reticulum | .(N)[^P][ST].. | - | - | 5.018e-03 |
| MOD_NEK2_1 | 226-231<br>[A]<br>342-347<br>[A]<br>347-352<br>[A]<br>388-393<br>[A] | -<br>-<br>-<br>- | NEK2 phosphorylation motif with preferred Phe, Leu or Met in the -3 position to compensate for less favorable residues in the +1 and +2 position. | centrosome, Ndc80 complex, condensed nuclear chromosome outer kinetochore, cytosol, nucleus | [FLM][^P][^P]([ST])[^DEP][^DE] | - | - | 9.798e-03 |
| MOD_NEK2_2 | 272-277<br>[A] | - | NEK2 phosphorylation motif with specific set of residues in the +1 and +2 position to compensate for less favorable residues in the -3 position. | centrosome, Ndc80 complex, condensed nuclear chromosome outer kinetochore, cytosol, nucleus | [WYPCAG][^P][^P]([ST])[IFCVML][KRHYF] | - | - | 1.295e-03 |
| MOD_PIKK_1 | 137-143<br>[A] | - | (ST)Q motif which is phosphorylated by PIKK | nucleus | ...([ST])Q.. | - | - | 9.230e-03 |

| | | | | | | | | |
|---|---|---|---|---|---|---|---|---|
| | | | family members. | | | | | |
| MOD_PKA_2 | 194-200 [A] | - | Secondary preference for PKA-type AGC kinase phosphorylation. | nucleus, cAMP-dependent protein kinase complex, cytosol | .R.([ST])[^P].. | - | - | 9.458e-03 |
| MOD_Plk_1 | 239-245 [A] 342-348 [A] 366-372 [A] | - - - | Ser/Thr residue phosphorylated by the Plk1 kinase | centralspindlin complex, nucleus, spindle, gamma-tubulin complex, midbody, cytosol, kinetochore, spindle midzone, nuclear condensin complex, cleavage furrow, nucleoplasm, microtubule organizing center | .[DNE][^PG][ST](([FYILMVW]..)\|([^PEDGKN][FWYLIVM]).) | - | - | 7.674e-03 |
| MOD_Plk_4 | 93-99 [A] 300-306 [A] 342-348 [A] 384-390 [A] | - - - - | Ser/Thr residue phosphorylated by Plk4 | cleavage furrow, nucleus, centriolar satellite, pericentriolar material, cytosol, SCF ubiquitin ligase complex, gamma-tubulin ring complex, centriole | ..[^IRFW]([ST])[ILMVFWY][ILMVFWY]. | - | - | 6.019e-03 |
| MOD_ProDKin_1 | 298-304 [A] | - | Proline-Directed Kinase (e.g. MAPK) phosphorylation site in higher eukaryotes. | nucleus, cytosol | ...([ST])P.. | - | - | 1.543e-02 |
| TRG_ENDOCYTIC_2 | 305-308 [A] 383-386 [A] | - - | Tyrosine-based sorting signal responsible for the interaction with mu subunit of AP (Adaptor Protein) complex | plasma membrane, clathrin-coated endocytic vesicle, cytosol | Y..[LMVIF] | - | - | 2.587e-03 |
| TRG_ER_diArg_1 | 203-205 [A] | - | The di-Arg ER retention motif is defined by two consecutive arginine residues (RR) or with a single residue insertion (RXR). The motif is completed by an adjacent hydrophobic/arginine residue which may be on either side of the Arg pair. | cytosol, endoplasmic reticulum membrane, integral protein, ER-Golgi transport vesicle membrane, endoplasmic reticulum membrane, Golgi-ER transport vesicle membrane, rough endoplasmic reticulum, endoplasmic reticulum cisterna | ([LIVMFYWPR]R[^YFWDE]{0,1}R)\|(R[^YFWDE]{0,1}R[LIVMFYWPR]) | - | - | 5.369e-03 |
| TRG_LysEnd_APsAcLL_1 | 222-227 [A] 384-389 [A] 425-430 [A] | - - - | Sorting and internalisation signal found in the cytoplasmic juxta-membrane region of type I transmembrane proteins. Targets them from the Trans Golgi Network to the lysosomal-endosomal-melanosomal compartments. Interacts with adaptor protein (AP) complexes | Endocytic vesicle, cytosol | [DERQ]...L[LVI] | - | - | 2.758e-03 |
| TRG_NES_CRM1_1 | 144-157 [A] 371-384 [A] 372-384 [A] | - - - | Some proteins re-exported from the nucleus contain a Leucine-rich nuclear export signal (NES) binding to the CRM1 exportin protein. | nucleus, cytosol | ([DEQ].{0,1}[LIM].{2,3}[LIVMF][^P]{2,3}[LMVF].[LMIV].{0,3}[DE])\|([DE].{0,1}[LIM].{2,3}[LIVMF][^P]{2,3}[LMVF].[LMIV].{0,3}[DEQ]) | - | - | 7.626e-04 |

# Summary for sequence 'PtoHopQ1'.

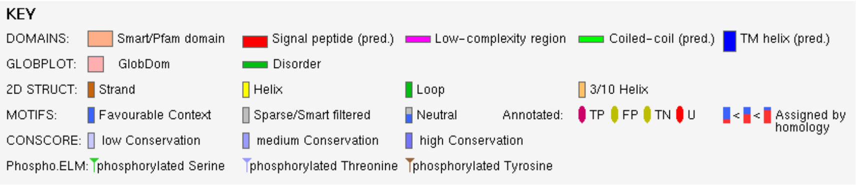


(Mouseover the matches for more details )

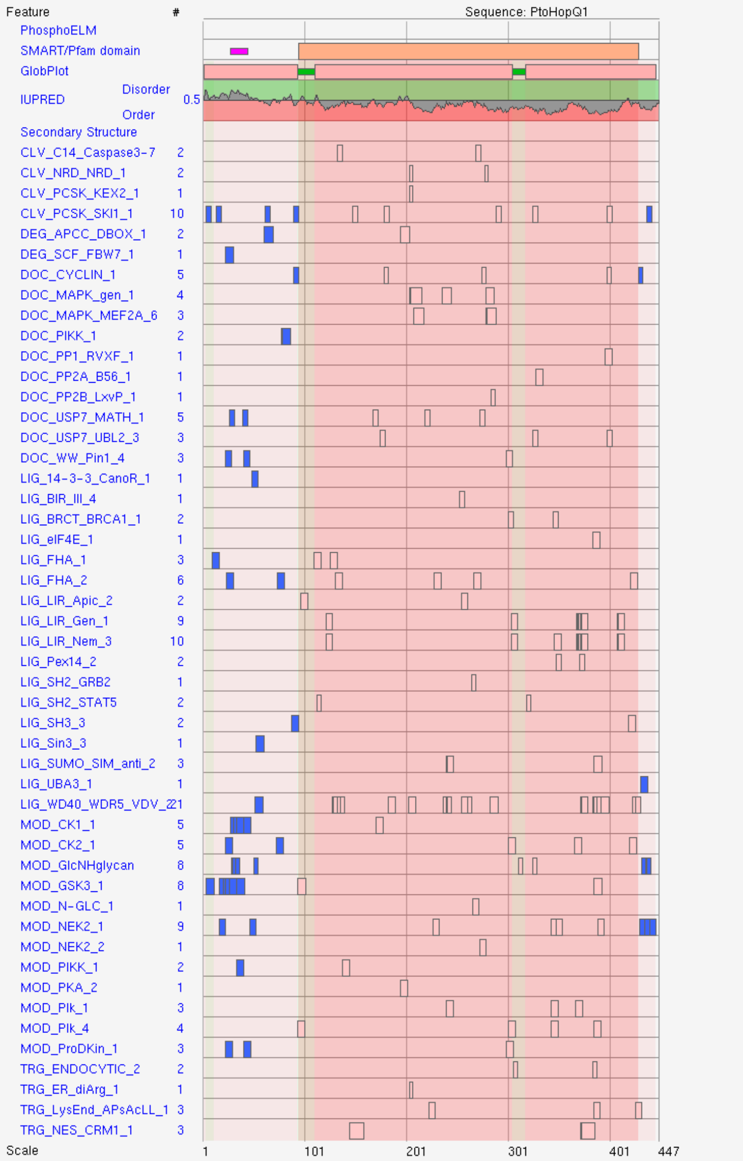


Either not enough data available to calculate a sequence alignment or the calculations haven't finished yet (in which case you can try to reload the page)...

## Filtering summary

**No user supplied cellular location.**
**User supplied taxon: root**

(An ELM is listed as filtered when all its matching instances have been filtered out.)

| | | Elms | Instances |
|---|---|---|---|
| **FILTERED BY:** | Species | 0 | 0 |
| | Cellular location (counts only those ELMs not already excluded by species.) | 0 | 0 |
| | Structural score (below medium threshold score) | 0 | 0 |
| | Smart (in a domain and no structural filter info available) | 28 | 124 |
| **TOTAL FILTERED:** | | 28 | 124 |
| **RETAINED BY:** | Smart (outside domain and no structural filter info available) | 21 | 49 |
| | Structural score (at or above medium threshold score) | 0 | 0 |
| **TOTAL RETAINED:** | | 21 | 49 |
| **TOTAL** | all found (before filtering) | 49 | 173 |

Query sequence:
>PtoHopQ1
MHRPITAGHTTSRLILDQSKQISRTPSESSAQSALSQQASMSSPVLERSKSAPALLTAAQ
RTMLAQVGACNAHLTSDENMAINELRSHKPLLPKDTWFFTDPNKDPDDVVTYTLGKQLQA
EGFVHITDVVATLGDAEVRSQRAEMAKGVFNKLELHDVHVSRGRDYAMNSLQSKEHAKFL
LEGHALRAGPGEIHRDSLQDMSRRLARAPHGVGIVVIAGMSDINALITTCPDMVRERVDD
ITIMGGVEPLKDADGFVQPDARAYNNATDMDAARSLYRKAQELGIPLRIVTKEAAYKTAV
SPSFYEGIAGSGHPVGHYLRDVQKSALKGLWEGIQAGLLPGLDDSWFFRTFMPNAQIEAA
QLDKNKESSFEDIWPKVTKLNLYDPLTLLASVPGAAKLLFKPKAIHTEGFGVVEQVGPDD
VTHPEKAKLLMSALAKSALVQSTVAPD

## Globular domains/ TM domains and signal peptide detected by the SMART server

| Domain | Start | End |
|---|---|---|
| Pfam:IU_nuc_hydro | 94 | 427 |

## Results of ELM motif search after globular domain filtering, structural filtering and context filtering.

**Matches falling inside globular protein domains are excluded from this list unless having an acceptable structural score (if the structural filter (BETA version) is applicable). If the structural filter (BETA version)is applicable it is possible to view these structures with Jmol**

| Elm Name | Instances (Matched Sequence) | Positions | View in Jmol | Elm Description | Cell Compartment | Pattern | PHI-Blast Instance Mapping | Structural Filter Info | Probability |
|---|---|---|---|---|---|---|---|---|---|
| CLV_PCSK_SKI1_1 | RPITA<br>RLILD<br>RTMLA<br>KPLLP<br>KSALV | 3-7 [A]<br>13-17 [A]<br>61-65 [A]<br>89-93 [A]<br>436-440 [A] | -<br>-<br>-<br>-<br>- | Subtilisin/kexin isozyme-1 (SKI1) cleavage site ([RK]-X-[hydrophobic]-[LTKF]-\|-X). | extracellular, Golgi apparatus, endoplasmic reticulum lumen, endoplasmic reticulum | [RK].[AILMFV][LTKF]. | - | - | 6.821e-03 |
| DEG_APCC_DBOX_1 | QRTMLAQVG | 60-68 [A] | - | An RxxL-based motif that binds to the Cdh1 and Cdc20 components of APC/C thereby targeting the protein for destruction in a cell cycle dependent manner | nucleus, cytosol | .R..L..[LIVM]. | - | - | 7.677e-04 |
| DEG_SCF_FBW7_1 | ISRTPSES | 22-29 [A] | - | The TPxxS phospho-dependent degron binds the FBW7 F box proteins of the SCF (Skp1_Cullin-Fbox) complex. | nucleus, cytosol | [LIVMP].{0,2}(T)P..([ST]) | - | - | 7.138e-04 |
| DOC_CYCLIN_1 | KPLLP<br>KLLM | 89-93 [A]<br>428-431 [A] | -<br>- | Substrate recognition site that interacts with cyclin and thereby increases phosphorylation by cyclin/cdk complexes. Predicted proteins should have a CDK phosphorylation site. Also used by cyclin/cdk inhibitors. | nucleus, cytosol | [RK].L.{0,1}[FYLIVMP] | - | - | 5.324e-03 |
| DOC_PIKK_1 | DENMAINEL<br>ENMAINEL | 77-85 [A]<br>78-85 [A] | -<br>- | DOC_PIKK_1 motif is located in the C terminus of Nbs1 and its homologues and interacts with PIKK family members. | nucleus | [DEN][DEN].{2,3}[ILMVA][DEN][DEN]L | - | - | 3.129e-05 |
| DOC_USP7_MATH_1 | PSESS<br>ASMSS | 26-30 [A]<br>39-43 [A] | -<br>- | The USP7 MATH domain binding motif variant based on the MDM2 and p53 interactions. | nucleus | [PA][^P][^FYWIL]S[^P] | - | - | 1.239e-02 |
| DOC_WW_Pin1_4 | ISRTPS<br>SMSSPV | 22-27 [A]<br>40-45 [A] | -<br>- | The Class IV WW domain interaction motif is recognised primarily by the Pin1 phosphorylation-dependent prolyl isomerase. | cytosol, nucleus | ...([ST])P. | - | - | 1.543e-02 |
| LIG_14-3-3_CanoR_1 | RSKSAP | 48-53 [A] | - | Canonical Arg-containing phospho-motif mediating a strong interaction with 14-3-3 proteins. | cytosol, internal side of plasma membrane, nucleus | R[^DE]{0,2}[^DEPG]([ST])(([FWYLMV].)\|([^PRIKGN]P)\|([^PRIKGN].{2,4}[VILMFWYP])) | - | - | 4.477e-03 |

| | | | | | | | | | |
|---|---|---|---|---|---|---|---|---|---|
| LIG_FHA_1 | HTTSRLI | 9-15 [A] | - | Phosphothreonine motif binding a subset of FHA domains that show a preference for a large aliphatic amino acid at the pT+3 position. | nucleus | ..(T)..[ILV]. | - | - | 8.662e-03 |
| LIG_FHA_2 | SRTPSES<br>HLTSDEN | 23-29 [A]<br>73-79 [A] | -<br>- | Phosphothreonine motif binding a subset of FHA domains that have a preference for an acidic amino acid at the pT+3 position. | nucleus,<br>Replication fork | ..(T)..[DE]. | - | - | 8.286e-03 |
| LIG_SH3_3 | SHKPLLP | 87-93 [A] | - | This is the motif recognized by those SH3 domains with a non-canonical class I recognition specificity | plasma membrane,<br>focal adhesion,<br>cytosol | ...[PV]..P | - | - | 1.317e-02 |
| LIG_Sin3_3 | APALLTAA | 52-59 [A] | - | Motif interacts with PAH2 domain in the Sin3 scaffold protein (not mad or sp-1 like). | nucleus | [FA].[LA][LV][LVI]..[AM] | - | - | 2.197e-05 |
| LIG_UBA3_1 | LMSALAK | 430-436 [A] | - | UBA3 adenylation domain binding motif variant based on the UBE2M and UBE2F interactions. | nucleus | [ILM][ILMF].{1,2}[ILM].{0,4}K | - | - | 1.196e-03 |
| LIG_WD40_WDR5_VDV_2 | SAPALLTA | 51-58 [A] | - | Fungi-specific variant of the WDR5-binding motif that binds to a cleft between blades 5 and 6 of the WD40 repeat domain of WDR5, opposite of the Win motif-binding site, to mediate assembly of histone modification complexes. | nucleus,<br>histone methyltransferase complex | [EDSTY].{0,4}[VIPLA][TSDEKR][ILVA] | - | - | 4.678e-02 |
| MOD_CK1_1 | SESSAQS<br>SAQSALS<br>SALSQQA<br>SMSSPVL | 27-33 [A]<br>30-36 [A]<br>33-39 [A]<br>40-46 [A] | -<br>-<br>-<br>- | CK1 phosphorylation site | nucleus,<br>cytosol | S..([ST])... | - | - | 1.704e-02 |
| MOD_CK2_1 | ISRTPSE<br>AHLTSDE | 22-28 [A]<br>72-78 [A] | -<br>- | CK2 phosphorylation site | nucleus,<br>protein kinase CK2 complex,<br>cytosol | ...([ST])..E | - | - | 1.457e-02 |
| MOD_GlcNHglycan | ESSAQ<br>SSAQ<br>QSAL<br>KSAP<br>MSAL<br>KSAL | 28-32 [A]<br>29-32 [A]<br>32-35 [A]<br>50-53 [A]<br>431-434 [A]<br>436-439 [A] | -<br>-<br>-<br>-<br>-<br>- | Glycosaminoglycan attachment site | extracellular,<br>Golgi apparatus | [ED]{0,3}.(S)[GA]. | - | - | 1.792e-02 |
| MOD_GSK3_1 | RPITAGHT<br>LDQSKQIS<br>KQISRTPS<br>ISRTPSES<br>PSESSAQS<br>SALSQQAS | 3-10 [A]<br>16-23 [A]<br>20-27 [A]<br>22-29 [A]<br>26-33 [A]<br>33-40 [A] | -<br>-<br>-<br>-<br>-<br>- | GSK3 phosphorylation recognition site | nucleus,<br>cytosol | ...([ST])...[ST] | - | - | 2.679e-02 |
| MOD_NEK2_1 | LDQSKQ<br>LERSKS<br>LLMSAL<br>LAKSAL<br>LVQSTV | 16-21 [A]<br>46-51 [A]<br>429-434 [A]<br>434-439 [A]<br>439-444 [A] | -<br>-<br>-<br>-<br>- | NEK2 phosphorylation motif with preferred Phe, Leu or Met in the -3 position to compensate for less favorable residues in the +1 and +2 position. | centrosome,<br>Ndc80 complex,<br>condensed nuclear chromosome outer kinetochore,<br>cytosol,<br>nucleus | [FLM][^P][^P]([ST])[^DEP][^DE] | - | - | 9.798e-03 |
| MOD_PIKK_1 | SALSQQA | 33-39 [A] | - | (ST)Q motif which is phosphorylated by PIKK family members. | nucleus | ...([ST])Q.. | - | - | 9.230e-03 |
| MOD_ProDKin_1 | ISRTPSE<br>SMSSPVL | 22-28 [A]<br>40-46 [A] | -<br>- | Proline-Directed Kinase (e.g. MAPK) phosphorylation site in higher eukaryotes. | nucleus,<br>cytosol | ...([ST])P.. | - | - | 1.543e-02 |

- **List of excluded ELMs falling inside SMART/PFAM domains and/or scoring poorly with the structural filter (if**

**applicable).**

**Matches in this list are only likely to be of interest if they are in accessible surface-exposed loops. Motif matches buried in stably folded cores of globular domains are not plausible candidates.**
**If the structural filter (BETA version) is applicable it is possible to view these structures with Jmol. For more info consult the PDB structure entry used for structure filtering or the SMART or PFAM entries for useful links to solved 3D structures.**

| Elm Name | Positions | View in Jmol | Elm Description | Cell Compartment | Pattern | PHI-Blast Instance Mapping | Structural Filter Info | Probability |
|---|---|---|---|---|---|---|---|---|
| CLV_C14_Caspase3-7 | 132-136 [A] 268-272 [A] | - - | Caspase-3 and Caspase-7 cleavage site. | nucleus, cytosol | [DSTE][^P][^DEWHFYC]D[GSAN] | - | - | 3.094e-03 |
| CLV_NRD_NRD_1 | 203-205 [A] 277-279 [A] | - - | N-Arg dibasic convertase (NRD/Nardilysin) cleavage site (X-\|-R-K or R-\|-R-X). | extracellular, Golgi apparatus, cell surface | (.RK)\|(RR[^KR]) | - | - | 7.465e-03 |
| CLV_PCSK_KEX2_1 | 203-205 [A] | - | Yeast kexin 2 cleavage site (K-R-\|-X or R-R-\|-X). | extracellular, Golgi apparatus | [KR]R. | - | - | 7.973e-03 |
| CLV_PCSK_SKI1_1 | 147-151 [A] 178-182 [A] 288-292 [A] 324-328 [A] 397-401 [A] | - - - - - | Subtilisin/kexin isozyme-1 (SKI1) cleavage site ([RK]-X-[hydrophobic]-[LTKF]-\|-X). | extracellular, Golgi apparatus, endoplasmic reticulum lumen, endoplasmic reticulum | [RK].[AILMFV][LTKF]. | - | - | 6.821e-03 |
| DEG_APCC_DBOX_1 | 194-202 [A] | - | An RxxL-based motif that binds to the Cdh1 and Cdc20 components of APC/C thereby targeting the protein for destruction in a cell cycle dependent manner | nucleus, cytosol | .R..L..[LIVM]. | - | - | 7.677e-04 |
| DOC_CYCLIN_1 | 178-181 [A] 274-277 [A] 397-400 [A] | - - - | Substrate recognition site that interacts with cyclin and thereby increases phosphorylation by cyclin/cdk complexes. Predicted proteins should have a CDK phosphorylation site. Also used by cyclin/cdk inhibitors. | nucleus, cytosol | [RK].L.{0,1}[FYLIVMP] | - | - | 5.324e-03 |
| DOC_MAPK_gen_1 | 203-214 [A] 204-214 [A] 235-243 [A] 278-285 [A] | - - - - | MAPK interacting molecules (e.g. MAPKKs, substrates, phosphatases) carry docking motif that help to regulate specific interaction in the MAPK cascade. The classic motif approximates (R/K)xxxx#x# where # is a hydrophobic residue. | nucleus, cytosol | [KR]{0,2}[KR].{0,2}[KR].{2,4}[ILVM].[ILVF] | - | - | 4.324e-03 |
| DOC_MAPK_MEF2A_6 | 207-216 [A] 278-287 [A] 279-287 [A] | - - - | A kinase docking motif that mediates interaction towards the ERK1/2 and p38 subfamilies of MAP kinases. | cytosol, Transcription factor complex, nucleus | [RK].{2,4}[LIVMP].[LIV].[LIVMF] | - | - | 2.584e-03 |
| DOC_PP1_RVXF_1 | 395-401 [A] | - | Protein phosphatase 1 catalytic subunit (PP1c) interacting motif binds targeting proteins that dock to the substrate for dephosphorylation. The motif defined is [RK]{0,1}[VI][^P][FW]. | nucleus, protein phosphatase type 1 complex, cytosol | ..[RK].{0,1}[VIL][^P][FW]. | - | - | 8.301e-04 |

| | | | | | | | | |
|---|---|---|---|---|---|---|---|---|
| DOC_PP2A_B56_1 | 327-333 [A] | - | Docking site required for the regulatory subunit B56 of PP2A for protein dephosphorylation. | nucleus, kinetochore, chromosome, centromeric region, cytosol | ([LMFYWIC]..I.E)\|(L..[IVLWC].E). | - | - | 1.458e-03 |
| DOC_PP2B_LxvP_1 | 283-286 [A] | - | Docking motif in calcineurin substrates that binds at the interface of the catalytic CNA and regulatory CNB subunits. | cytosol, calcineurin complex, nucleus | L.[LIVAPM]P | - | - | 2.296e-03 |
| DOC_USP7_MATH_1 | 167-171 [A] 218-222 [A] 272-276 [A] | - - - | The USP7 MATH domain binding motif variant based on the MDM2 and p53 interactions. | nucleus | [PA][^P][^FYWIL]S[^P] | - | - | 1.239e-02 |
| DOC_USP7_UBL2_3 | 174-178 [A] 324-328 [A] 397-401 [A] | - - - | The USP7 CTD domain binding motif variant based on the ICP0 and DNMT1 interactions | nucleus | K...K | - | - | 3.742e-03 |
| DOC_WW_Pin1_4 | 298-303 [A] | - | The Class IV WW domain interaction motif is recognised primarily by the Pin1 phosphorylation-dependent prolyl isomerase. | cytosol, nucleus | ...([ST])P. | - | - | 1.543e-02 |
| LIG_BIR_III_4 | 252-256 [A] | - | These IBMs are found in the N-terminal regions of arthropodal caspase subunits where they mediate the inhibition of activated caspases by binding to conserved surface grooves on type III BIR domains of Inhibitor of Apoptosis Proteins (IAPs). | mitochondrion, cytosol | DA.G. | - | - | 2.754e-04 |
| LIG_BRCT_BRCA1_1 | 300-304 [A] 344-348 [A] | - - | Phosphopeptide motif which directly interacts with the BRCT (carboxy-terminal) domain of the Breast Cancer Gene BRCA1 with low affinity | nucleus, BRCA1-BARD1 complex | .(S)..F | - | - | 1.912e-03 |
| LIG_eIF4E_1 | 383-389 [A] | - | Motif binding to the dorsal surface of eIF4E. | cytosol | Y....L[VILMF] | - | - | 1.891e-04 |
| LIG_FHA_1 | 109-115 [A] 125-131 [A] | - - | Phosphothreonine motif binding a subset of FHA domains that show a preference for a large aliphatic amino acid at the pT+3 position. | nucleus | ..(T)..[ILV]. | - | - | 8.662e-03 |
| LIG_FHA_2 | 130-136 [A] 227-233 [A] 266-272 [A] 420-426 [A] | - - - - | Phosphothreonine motif binding a subset of FHA domains that have a preference for an acidic amino acid at the pT+3 position. | nucleus, Replication fork | ..(T)..[DE]. | - | - | 8.286e-03 |
| LIG_LIR_Apic_2 | 96-102 [A] 254-259 [A] | - - | Apicomplexa specific variant of the canonical LIR motif that binds to Atg8 protein family members to mediate processes involved in autophagy. | cytosol, cytoplasmic side of late endosome membrane | [EDST].{0,2}[WFY]..P | - | - | 3.371e-03 |
| LIG_LIR_Gen_1 | 121-126 [A] 303-308 [A] 367-373 [A] | - - - - - - | | cytosol, cytoplasmic side of late endosome membrane | [EDST].{0,2}[WFY]..[ILV] | - | - | 5.200e-03 |

| | | | | | | | | |
|---|---|---|---|---|---|---|---|---|
| | 368-373 [A]<br>369-373 [A]<br>371-377 [A]<br>372-377 [A]<br>407-413 [A]<br>408-413 [A] | -<br>-<br>- | Canonical LIR motif that binds to Atg8 protein family members to mediate processes involved in autophagy. | | | | | |
| LIG_LIR_Nem_3 | 121-126 [A]<br>303-308 [A]<br>345-351 [A]<br>367-373 [A]<br>368-373 [A]<br>369-373 [A]<br>371-377 [A]<br>372-377 [A]<br>407-413 [A]<br>408-413 [A] | -<br>-<br>-<br>-<br>-<br>-<br>-<br>-<br>-<br>- | Nematode-specific variant of the canonical LIR motif that binds to Atg8 protein family members to mediate processes involved in autophagy. | cytosol, cytoplasmic side of late endosome membrane | [EDST].{0,2}[WFY]..[ILVFY] | - | - | 6.362e-03 |
| LIG_Pex14_2 | 347-351 [A]<br>370-374 [A] | -<br>- | Fxxx[WF] motifs are present in Pex19 and S. cerevisiae Pex5 cytosolic receptors that bind to peroxisomal membrane docking member, Pex14 | cytosol, peroxisome, glycosome | F...[WF] | - | - | 4.628e-04 |
| LIG_SH2_GRB2 | 264-267 [A] | - | GRB2-like Src Homology 2 (SH2) domains binding motif. | cytosol | (Y).N. | - | - | 4.787e-04 |
| LIG_SH2_STAT5 | 112-115 [A]<br>318-321 [A] | -<br>- | STAT5 Src Homology 2 (SH2) domain binding motif. | cytosol | (Y)[VLTFIC].. | - | - | 3.296e-03 |
| LIG_SH3_3 | 418-424 [A] | - | This is the motif recognized by those SH3 domains with a non-canonical class I recognition specificity | plasma membrane, focal adhesion, cytosol | ...[PV]..P | - | - | 1.317e-02 |
| LIG_SUMO_SIM_anti_2 | 239-245 [A]<br>240-245 [A]<br>384-391 [A] | -<br>-<br>- | Motif for the antiparallel beta augmentation mode of non-covalent binding to SUMO protein. | nucleus, PML body, nuclear body | [DEST]{1,10}.{0,1}[VIL][DESTVILMA][VIL][VILM].[DEST]{0,5} | - | - | 2.349e-03 |
| LIG_WD40_WDR5_VDV_2 | 127-133 [A]<br>128-133 [A]<br>132-138 [A]<br>135-138 [A]<br>182-188 [A]<br>202-208 [A]<br>236-243 [A]<br>239-243 [A]<br>240-243 [A]<br>254-261 [A]<br>260-263 [A] | -<br>-<br>-<br>-<br>-<br>-<br>-<br>-<br>-<br>-<br>-<br>-<br>-<br>-<br>-<br>-<br>-<br>-<br>-<br>- | Fungi-specific variant of the WDR5-binding motif that binds to a cleft between blades 5 and 6 of the WD40 repeat domain of WDR5, opposite of the | nucleus, histone methyltransferase complex | [EDSTY].{0,4}[VIPLA][TSDEKR][ILVA] | - | - | 4.678e-02 |

| | | | | | | | | |
|---|---|---|---|---|---|---|---|---|
| | 282-289 [A] 371-377 [A] 372-377 [A] 383-388 [A] 384-388 [A] 387-392 [A] 391-398 [A] 422-429 [A] 425-429 [A] | | Win motif-binding site, to mediate assembly of histone modification complexes. | | | | | |
| MOD_CK1_1 | 170-176 [A] | - | CK1 phosphorylation site | nucleus, cytosol | S..([ST])... | - | - | 1.704e-02 |
| MOD_CK2_1 | 300-306 [A] 365-371 [A] 419-425 [A] | - - - | CK2 phosphorylation site | nucleus, protein kinase CK2 complex, cytosol | ...([ST])..E | - | - | 1.457e-02 |
| MOD_GlcNHglycan | 310-313 [A] 324-327 [A] | - - | Glycosaminoglycan attachment site | extracellular, Golgi apparatus | [ED]{0,3}.(S)[GA]. | - | - | 1.792e-02 |
| MOD_GSK3_1 | 93-100 [A] 384-391 [A] | - - | GSK3 phosphorylation recognition site | nucleus, cytosol | ...([ST])...[ST] | - | - | 2.679e-02 |
| MOD_N-GLC_1 | 265-270 [A] | - | Generic motif for N-glycosylation. It was shown that Trp, Asp, and Glu are uncommon before the Ser/Thr position. Efficient glycosylation usually occurs when ~60 residues or more separate the glycosylation acceptor site from the C-terminus. | extracellular, Golgi apparatus, endoplasmic reticulum | .(N)[^P][ST].. | - | - | 5.018e-03 |
| MOD_NEK2_1 | 226-231 [A] 342-347 [A] 347-352 [A] 388-393 [A] | - - - - | NEK2 phosphorylation motif with preferred Phe, Leu or Met in the -3 position to compensate for less favorable residues in the +1 and +2 position. | centrosome, Ndc80 complex, condensed nuclear chromosome outer kinetochore, cytosol, nucleus | [FLM][^P][^P]([ST])[^DEP][^DE] | - | - | 9.798e-03 |
| MOD_NEK2_2 | 272-277 [A] | - | NEK2 phosphorylation motif with specific set of residues in the +1 and +2 position to compensate for less favorable residues in the -3 position. | centrosome, Ndc80 complex, condensed nuclear chromosome outer kinetochore, cytosol, nucleus | [WYPCAG][^P][^P]([ST])[IFCVML][KRHYF] | - | - | 1.295e-03 |
| MOD_PIKK_1 | 137-143 [A] | - | (ST)Q motif which is phosphorylated by PIKK family members. | nucleus | ...([ST])Q.. | - | - | 9.230e-03 |
| MOD_PKA_2 | 194-200 [A] | - | Secondary preference for PKA-type AGC kinase phosphorylation. | nucleus, cAMP-dependent protein kinase complex, cytosol | .R.([ST])[^P].. | - | - | 9.458e-03 |
| MOD_Plk_1 | 239-245 [A] 342-348 [A] 366-372 [A] | - - - | | centralspindlin complex, nucleus, spindle, gamma-tubulin complex, | .[DNE][^PG][ST](([FYILMVW]..)\|([^PEDGKN][FWYLIVM]).) | - | - | 7.674e-03 |

| | | | | | | | | |
|---|---|---|---|---|---|---|---|---|
| | | | Ser/Thr residue phosphorylated by the Plk1 kinase | midbody, cytosol, kinetochore, spindle midzone, nuclear condensin complex, cleavage furrow, nucleoplasm, microtubule organizing center | | | | |
| MOD_Plk_4 | 93-99 [A] 300-306 [A] 342-348 [A] 384-390 [A] | - - - - | Ser/Thr residue phosphorylated by Plk4 | cleavage furrow, nucleus, centriolar satellite, pericentriolar material, cytosol, SCF ubiquitin ligase complex, gamma-tubulin ring complex, centriole | ..[^IRFW]([ST])[ILMVFWY][ILMVFWY]. | - | - | 6.019e-03 |
| MOD_ProDKin_1 | 298-304 [A] | - | Proline-Directed Kinase (e.g. MAPK) phosphorylation site in higher eukaryotes. | nucleus, cytosol | ...([ST])P.. | - | - | 1.543e-02 |
| TRG_ENDOCYTIC_2 | 305-308 [A] 383-386 [A] | - - | Tyrosine-based sorting signal responsible for the interaction with mu subunit of AP (Adaptor Protein) complex | plasma membrane, clathrin-coated endocytic vesicle, cytosol | Y..[LMVIF] | - | - | 2.587e-03 |
| TRG_ER_diArg_1 | 203-205 [A] | - | The di-Arg ER retention motif is defined by two consecutive arginine residues (RR) or with a single residue insertion (RXR). The motif is completed by an adjacent hydrophobic/arginine residue which may be on either side of the Arg pair. | cytosol, endoplasmic reticulum membrane, integral protein, ER-Golgi transport vesicle membrane, endoplasmic reticulum membrane, Golgi-ER transport vesicle membrane, rough endoplasmic reticulum, endoplasmic reticulum cisterna | ([LIVMFYWPR]R[^YFWDE]{0,1}R)\|(R[^YFWDE]{0,1}R[LIVMFYWPR]) | - | - | 5.369e-03 |
| TRG_LysEnd_APsAcLL_1 | 222-227 [A] 384-389 [A] 425-430 [A] | - - - | Sorting and internalisation signal found in the cytoplasmic juxta-membrane region of type I transmembrane proteins. Targets them from the Trans Golgi Network to the lysosomal-endosomal-melanosomal compartments. Interacts with adaptor protein (AP) complexes | Endocytic vesicle, cytosol | [DERQ]...L[LVI] | - | - | 2.758e-03 |
| TRG_NES_CRM1_1 | 144-157 [A] 371-384 [A] 372-384 [A] | - - - | Some proteins re-exported from the nucleus contain a Leucine-rich nuclear export signal (NES) binding to the CRM1 exportin protein. | nucleus, cytosol | ([DEQ].{0,1}[LIM].{2,3}[LIVMF][^P]{2,3}[LMVF].[LMIV].{0,3}[DE])\|([DE].{0,1}[LIM].{2,3}[LIVMF][^P]{2,3}[LMVF].[LMIV].{0,3}[DEQ]) | - | - | 7.626e-04 |

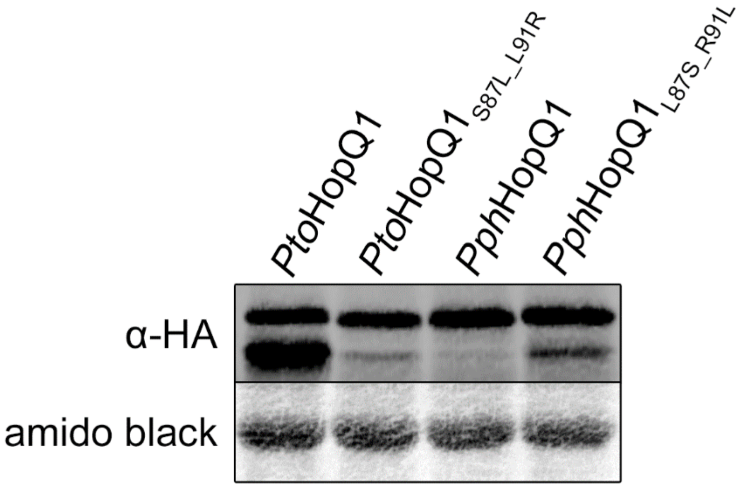


**Supplementary Figure 3** Presence of S87 and L91 increases HopQ1 susceptibility to proteolytic cleavage. HA-tagged HopQ1 variants were transiently expressed in Arabidopsis protoplasts. Fourteen hours after transformation, crude protein extracts were prepared, resolved by SDS-PAGE and subjected to immunoblot analysis using specific anti HA-antibodies. Amido black staining of the membranes was used to demonstrate equal loading. The experiment was performed four times with similar results.
